# Facile Determination of Aluminum Content in Industrial Brine by Investigating the Effects of Buffer Systems

**DOI:** 10.1002/open.202400038

**Published:** 2024-09-03

**Authors:** Benjámin Csorba, László Farkas, Marcell Csécsi, László T. Mika, Iván L. Gresits

**Affiliations:** ^1^ Department of Chemical and Environmental Process Engineering Faculty of Chemical Technology and Biotechnology Budapest University of Technology and Economics Műegyetem rkp. 3. H-1111 Budapest Hungary; ^2^ Process Technology Support BorsodChem Ltd. Bolyai tér 1. H-3700 Kazincbarcika Hungary

**Keywords:** Aluminum, Eriochrome cyanine R, Good's buffers, Industrial analytics, UV/Vis spectroscopy

## Abstract

The aluminum content of concentrated (27 wt%) sodium chloride solutions could be crucial for large‐scale chlor‐alkali‐based industries applying membrane cell electrolysis. Thus, a facile method which enables a fast and reliable protocol to determine the Al content of these solutions on ppb scale in industrial environments is fundamentally important. It was demonstrated that the increased sensitivity of colorful Al‐ECR (eriochrome cyanine R) complex by the use of a cationic surfactant and specific biological buffers could effectively indicate the Al content in an extended pH interval of a concentrated saline medium under industrial conditions. The dependence of the analytical protocol on pH, temperature, time, wavelength, and the salinity of the medium was investigated. It was shown that the absorbance‐based measurements of the solution should be performed at least 2–4 h after its preparation. By applying the selected two Good's buffers (HEPES: 4‐(2‐hydroxyethyl)‐1‐piperazineethanesulfonic acid, MOPS: 3‐(N‐morpholino)‐propanesulfonic acid) and Tris (tris(hydroxymethyl)aminomethane), 32.8–38.1 % increase in the sensitivity was achieved for saturated NaCl solutions. Moreover, the limits of detection and quantification (LOD, LOQ) were also lowered by 19.0–29.8 %, and the salinity dependence of the calibration was also reduced.

## Graphical abstract

The trace aluminum content of concentrated (27 wt%) sodium chloride solutions is crucial for chlor‐alkali industry. The increased sensitivity of colorful Al‐ECR (eriochrome cyanine R) complex by the use of a cationic surfactant and specific biological buffers could effectively indicate the Al content. The effect of pH, temperature, time, wavelength, and the salinity was investigated.

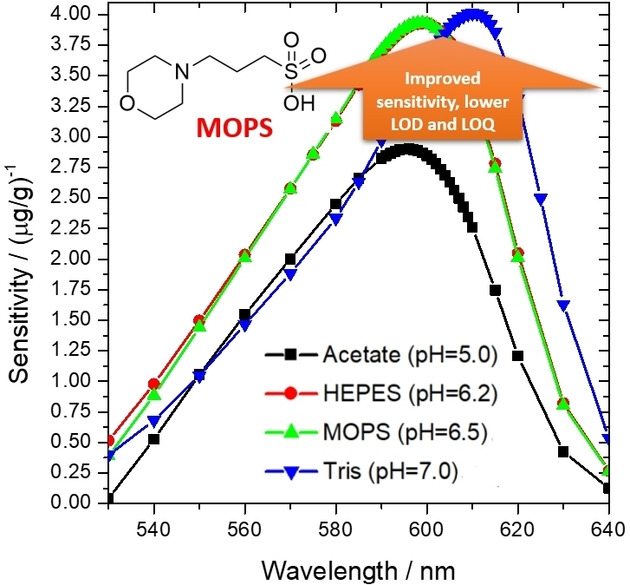

## Introduction

Reliable, accurate, and even facile measurement protocol for the determination of trace aluminum content in the ppb concentration range from various matrices is fundamentally important in several fields, from the chemical industry to healthcare.[^1^, ^2^, ^3^, ^4^, ^5^, ^6^, ^7^, ^8^, ^9^] The precise Al content measurements are crucial for the environmentally benign mercury‐free membrane cell‐based technology for chlor‐alkali electrolysis, requiring a very pure brine as a feedstock. The concentration level of its trace elements, such as metals (Ca, Mg, Al, Fe, Ba, Sr) and silicon, are maximized in the order of ppb because they can irreversibly damage the membrane of the electrolysis cell[^3^, ^10^, ^11^, ^12^, ^13^, ^14^] resulting in serious operational and economic concerns. For example, the significant electric energy needs can increase with a decrease in the quality and quantity of the product.^[10]^ Thus, monitoring and removing the Al content of the brine is of utmost importance for these technologies. Consequently, the development of a potential facile and viable method with high sensitivity and lower detection limit for determining aluminum content in concentrated saline medium is highly desired.

**Figure 1:**
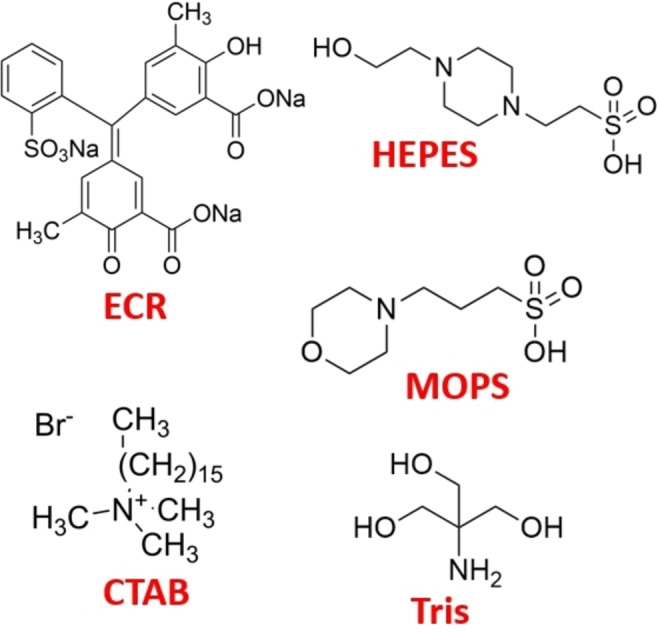
Molecular structure of ECR, CTAB and the examined biological buffers.

Although several methods for determining the trace amount of aluminum with sufficient accuracy have already been known, these methods usually require high investments and operating costs, for example, graphite furnace atomic absorption (GF‐AAS), inductively coupled plasma atomic emission (ICP‐AES) spectroscopy, or ^27^Al nuclear magnetic resonance (NMR) measurement.[^15^, ^16^, ^17^, ^18^] On the other hand, commonly used photometric techniques as analytical tools could serve as a promising alternative for accurately determining Al content in various matrices.[^19^, ^20^] These methods typically use specific organic complexing reagents that selectively form highly colorful complexes with Al assisting better detection parameters such as lower detection limit and/or higher sensitivity.[^21^, ^22^, ^23^, ^24^, ^25^, ^26^, ^27^, ^28^, ^29^, ^30^, ^31^, ^32^, ^33^, ^34^, ^35^, ^36^, ^37^, ^38^, ^39^, ^40^, ^41^, ^42^, ^43^, ^44^, ^45^, ^46^, ^47^, ^48^] Among these reagents, the bidentate ligand eriochrome cyanine R (ECR, shown on Figure 1) could be considered as superior complexing substance having a high molar absorption coefficient (*ϵ*) of 6.5×10^4^ Lmol^−1^cm^−1^.^[49]^ However, the species distribution of the different Al‐ECR complexes significantly depends on the pH, and the ECR/Al(III) molar ratio in the complexes could be a maximum of 3.[^50^, ^51^, ^52^, ^53^] The molar absorption coefficient could be further increased by adding different surfactants.[^19^, ^49^, ^54^, ^55^, ^56^, ^57^] In addition, it could also be facilitated by increasing the salt content of the solution, which is a key parameter in industrial brine. We demonstrated that the value of *ϵ* could be doubled (1.05×10^5^ Lmol^−1^cm^−1^) by the addition of cetyltrimethylammonium bromide (CTAB, shown on Figure 1) in acetic acid/sodium acetate buffer at a pH of 5 in concentrated saline medium. This method was able to detect the trace aluminum content in a dynamic range from 8.4 ppb to 690 ppb.^[58]^

Our previous study showed that the effect of pH was notable: the absorbance values measured for the same samples were the highest at a pH between 6.0–6.5 in the concentrated saline medium.^[58]^ Therefore, the replacement of acetic acid/sodium acetate buffer system can be proposed because it is unsuitable for adjusting pH values of 6 and above.

The selection of buffer systems should meet some crucial requirements as follows: (i) precipitation by either of the solution's components should be avoided, (ii) it should be inert (no reaction with components present), (iii) it does not absorb light around 600 nm, (iv) the acid dissociation constant must be adequate to application at pH=6–7, (v) it should have high‐water solubility minimizing the sample dilution.

These conditions largely coincide with the criteria of Good et al.^[59]^ which were formulated more than half a century ago as an objective for the development of buffers to be used in aqueous biological systems between pH from 6 to 8. In the second half of the twentieth century, 20 buffers corresponding to the above objectives were developed. Recently, these extensively used buffers are so‐called Good's buffers and are not limited to biological and/or biochemical utilization.[^60^, ^61^, ^62^, ^63^, ^64^] Beyond biological systems, ionic liquids and aqueous biphasic systems (ATPS or ABS) can also be established using Good's buffers.[^65^, ^66^, ^67^] The Good's buffers have also been found suitable to buffer media for reactions of gold nanoparticles[^68^, ^69^] and for pH imaging using hyperpolarized ^13^C NMR.^[70]^ Although Good's buffers are considered inert environments, they can form complexes with certain metals e. g. Cr(III), Fe(III), Mg(II), Ca(II), Mn(II), Co(II), Ni(II), Cu(II), Zn(II), Cd(II), Pb(II).[^60^, ^71^, ^72^, ^73^] This is a fundamental question, which, despite this, has been ignored in the studies of many researchers.^[60]^ It should also be noted that some Good's buffers could affect oxidation‐reduction processes, as reported by He et al.^[74]^ for the corrosion of elemental iron. However, any possibility of an oxidation reaction could be completely excluded, because the measurement of Al occurs in the highest oxidation state, in our cases. It should also be noted that the pH of the given buffers may vary depending on the temperature.^[75]^

Based on these key advantages of Good's buffers, herein we propose the application of 4‐(2‐hydroxyethyl)‐1‐piperazineethanesulfonic acid (HEPES), and 3‐(N‐morpholino)propanesulfonic acid (MOPS) buffers in our recently reported analytical tool for Al determination in the concentrated saline medium. The reason for our choice was that HEPES and MOPS did not, or only slightly, enter into a complex formation reaction with the metal ions examined in the literature.[^60^, ^76^] In addition, we also examined the tris(hydroxymethyl)aminomethane (Tris) buffer, which is also frequently used in biological systems, and its derivatives also include Good's buffers, but Tris itself is not a Good's buffer.^[77]^ The structures of the mentioned buffers are shown on Figure 1. However, when introducing a new buffer medium, the effect of time, temperature, pH and salinity on the method applicability have to be investigated in detail to deliver an optimum condition of the improved analytical tool.

## Results and Discussion

To improve the sensitivity of the proposed analytical method, the identification of an appropriate buffer system meeting the criteria noted above is crucial. Thus, several buffer systems were investigated for aluminum determination in the range of pH=6–7, first. Because of possible reactions including complex and insoluble precipitation formation with Al in the given pH range phosphate, tartarate, malonate, and citrate‐based buffers were discarded. It should be noted that malonate and citrate have limited applicability because the absorbance of the standard solution containing 400 ng/g aluminum remained below 0.1, which was significantly far from our recently reported acetate‐containing system.^[58]^ Consequently, in light of their critical intrinsic properties, Good's biological buffers, e. g. HEPES, MOPS, and Tris were subjected to our investigations.^[73]^

Our experiments established that all three biological buffers were suitable for the determination of trace amounts of dissolved aluminum in concentrated saline medium and the case of lower salinity. It was also shown that all three buffers could be kept in a homogeneous solution with CTAB, thereby reducing the number of solutions added to the sample from 3 (ECR, CTAB, buffer) to 2 (ECR, CTAB‐buffer). It should be noted that further reduction of required solutions is not possible, due to the spontaneous degradation of ECR and CTAB in a common assay solution. When different dosing sequences were performed, less than a 2 % difference in the measured absorbance was observed. Thus, the dosing sequence has no effect on the method's performance for all the tested biological buffers.

Based on previous[^49^, ^58^] as well as later detailed experiments, the measurement with the biological buffer systems can easily be performed as follows:

– Add ECR assay solution to the sample in a 1 : 6 mass ratio,
– Add the CTAB‐buffer solution of 1/3 of the weight of the initial sample to the system,
– Measure the absorbance of the sample after 2 (in case of MOPS) or 4 (in case of HEPES and Tris) hours, but on the same day (in the case of HEPES buffer, possibly the next day). Based on the dilution ratio and the previously taken calibration curve, the dissolved Al concentration of the sample can be specified.

**Figure 2:**
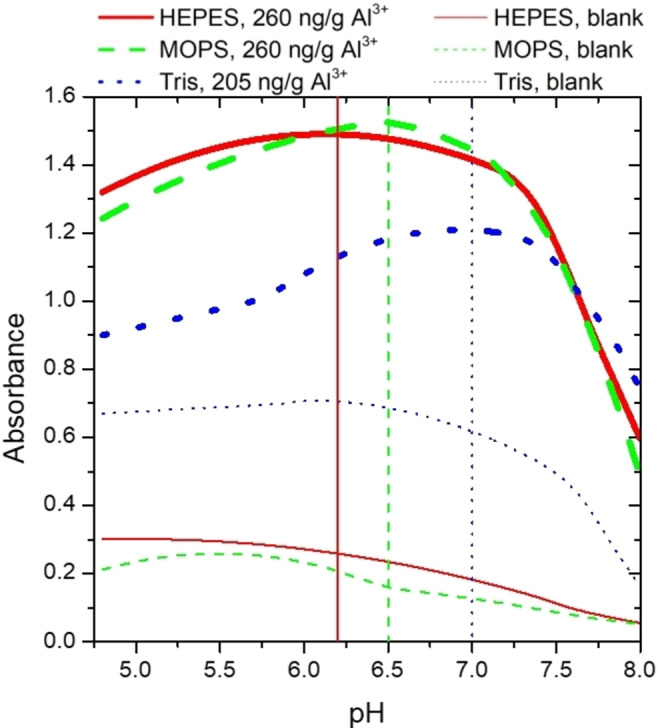
Effect of pH on the analytical signal, examining the saturated NaCl medium.

**Figure 3:**
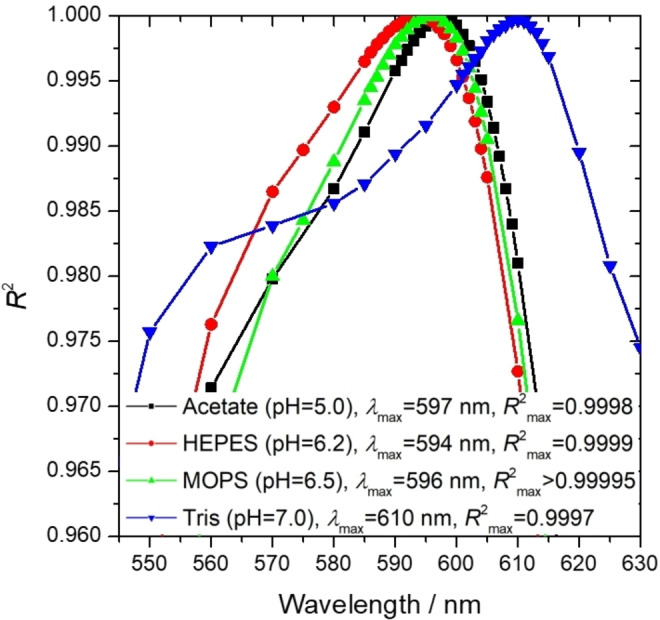
*R* ^2^ coefficient of the calibration line for each buffer system at optimal pH as a function of wavelength, examining the saturated NaCl medium. The source of the acetate buffer data is our own previous article.^[58]^

Since the pH value of the solution is critical for the proposed analytical method, subsequently the pH dependence of the analytical signal for each biological buffer was investigated and the optimum pH value, at which the blank‐corrected absorbance was maximal in given aluminum‐containing solutions, was determined. The pH values were adjusted between 3–10 by the addition of concentrated HCl solution or solid NaOH to minimize the possible effect of dilution and elimination of any trace ions of the system, which could affect the analysis via undesired side‐reactions. Figure 2. clearly shows that both pH values between 4.75–8.00 and the applied biological buffer have significant effects on the analytical signal. This may be due to the interactions of buffers with complexes.[^60^, ^72^, ^73^] The effect of pH on the analytical signal is possibly due to the pH dependence of the species distribution of ECR. In addition to the acid‐base properties of ECR, Shokrollahi et al.^[49]^ have investigated in details the composition of Al‐ECR complexes that can be formed at different pH values in a system with an ionic strength of 0.1 M. The different complexes could be present in the highly saline medium we investigated, in which even additional complexes could be formed. The spectra of the different complexes predominant at different pH values may also differ, resulting in a change in absorbance. This effect is already present in the absence of Al ions (as shown on Figure 3 for blank solutions). It was also reported that buffers, especially Tris, can form complexes with many metal ions. Thus, any interaction affecting the signal cannot be excluded in our cases.

The pH dependence was also examined for samples with different aluminum content for all three buffers. In the case of HEPES and MOPS using 260 ng/g Al concentration (we chose the value corresponding to the higher Al^3+^ concentration as optimal because at lower concentrations, the blank‐corrected curve is very flat), the optimal pH was found to be 6.2, and 6.5 respectively. For the Tris‐containing system, pH 7 was determined as optimum at 205 ng/g Al. In the case of MOPS buffer, no Al‐concentration dependence on optimal pH value, which can be highlighted as an advantage of this system, was observed in the range of 81.3 ng/g–260 ng/g. In addition, a further advantage of MOPS is that the optimal pH obtained is the closest to the p*K*_a_ value of the given buffer, contributing to the achievement of a higher buffer capacity than using Tris and HEPES.^[60]^ In the case of Tris, a higher absorbance of the blank solution as a disadvantage was found in the same pH range (Figure 2). Consequently, the Al content was adjusted to 205 ng/g to keep absorbance values below 2, according to the limitation of Lambert–Beer law.

**Figure 4:**
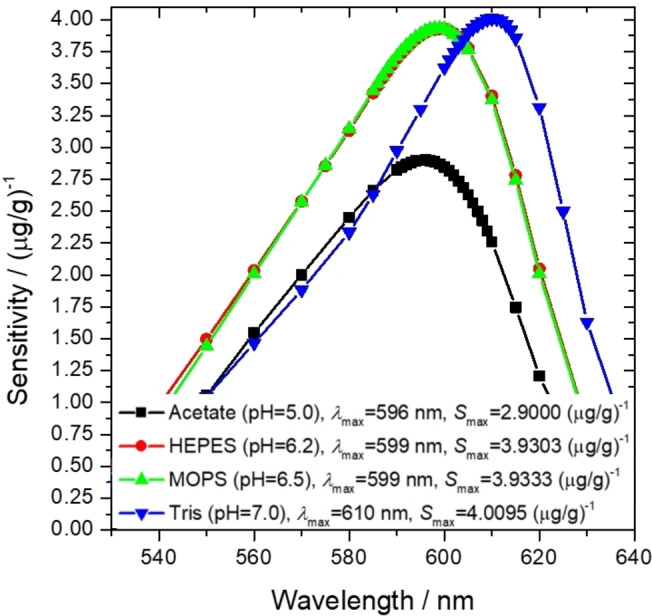
Sensitivities of the measurement for each buffer system at optimal pH as a function of wavelength, examining the saturated NaCl medium. The source of the acetate buffer data is our own previous article.^[58]^

The sensitivity of the Al^3+^ measurement could be improved if the pH was increased by 1–2 units compared to the optimal pH of 5 in the dilute medium.[^49^, ^58^] Accordingly, the wavelength dependence of the sensitivity and the *R* ^2^ coefficients of the calibration lines were also examined in the range of 400–800 nm. The results are summarized on Figure 3 and Figure 4. In all cases, the data refer to the measurement performed at the optimal pH (6.2 for HEPES, 6.5 for MOPS, 7.0 for Tris) for samples with a concentrated (27 wt% in the original brine) salt content.

It can be established that in the case of the concentrated saline medium, the sensitivity at optimal pHs could be increased for all buffers by 32.8–38.1 % compared to the acetate‐based system at pH=5. While the sensitivity of the measurement was increased significantly, the regression quality did not change and maintained *R* ^2^>0.9997. It can be assumed that the applied buffers either did not form a binary complex with Al^3+^ ions or their binding affinities were too weak to modify the effect of the Al‐ECR complex and to decrease the applicability of the measurements.

The optimal wavelength of the measurement could be identified according to two approaches: (i) it could be at the value of wavelength where the sensitivity of the measurement is maximal, or (ii) where the most accurate fitting can be made, e. g. the *R* ^2^ coefficient of the fitted line is maximal. Notably, while the optimal wavelength of the measurement in the case of MOPS and HEPES is similar to the value obtained for acetate buffer, it increases significantly in the case of Tris for both approaches.

**Figure 5:**
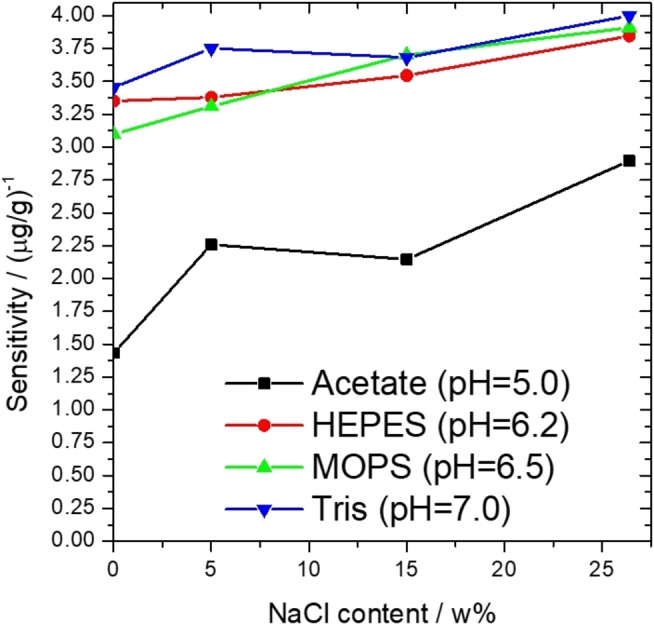
The sensitivity of the measurement as a function of the salt content of the sample in the specific buffer systems (the sensitivity data in the figure are 2/3 of the slopes of the calibration lines for the maximum *R* ^2^ coefficient). The source of the acetate buffer data is our own previous article.^[58]^

Subsequently, the influence of salinity on the sensitivity of the measurement was monitored for different buffer‐based systems. In acetate buffer, the sensitivity increased significantly at a pH of 5, even at relatively low (5 %) salinity compared to the result in NaCl‐free medium; moreover, further increase could be observed at concentrated solution (27 wt% in the original brine) reaching a total of 102 % growth compared to the NaCl‐free medium (Figure 5).^[58]^ The same tendency could be observed for Good's buffers and Tris at a pH of 6–7; however, this effect was less significant. In the case of HEPES, MOPS, and Tris, 14.8 %, 26.3 %, and 15.8 % increases were detected, respectively. The sensitivity‐salinity data compared to the data obtained for the acetate‐based system are summarized on Figure 5. It showed that the influence of salinity on sensitivity was less prevalent in buffers operating at higher pH. On the one hand, this is a disadvantage since a smaller improvement in sensitivity can be achieved by increasing the salt content. However, it can be considered as an advantage as well. If a specific measurement task does not require special accuracy, the measurement can be simplified, though it is not necessary to know the salt content of the sample since its effect on the parameters of the calibration line is much smaller than in the case of acetate buffer. It can also be concluded that while the salinity has minor effect on the wavelength associated with the most accurate fit, the wavelength associated with the highest sensitivity changes significantly depending on the salinity. More details can be found in the Supporting Information.

The formation and stability of the Al‐complex system under concentrated saline conditions are crucial to its robustness and viable applicability. Accordingly, the time‐dependent stability of the absorbance for the Good's buffers and Tris‐containing test solution was investigated in 0–72 h time window. It is, moreover, important to determine the maximum absorbance‐keeping time for each solution.

**Figure 6:**
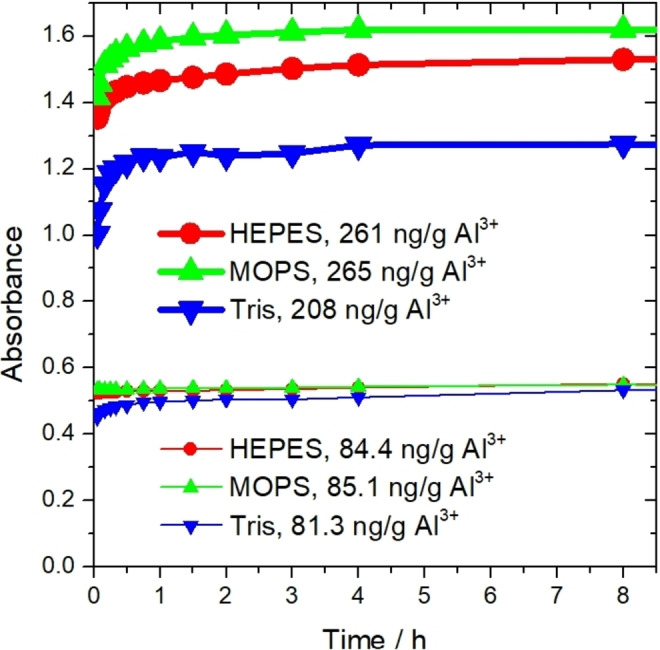
Time dependence of the analytical signal using different buffers, examining the saturated NaCl medium.

Examining the time‐dependence of the analytical signal revealed that reaching the signals’ maximum requires significantly more time for all new buffer systems than that of showed for acetate‐based system.^[58]^ It can be assumed that pH dependence of the complex stability constants and reaction rates has significant effect on reaching the equilibrium species distribution.^[49]^ The maximum analytical signal in HEPES buffer was formed in 8 h, but 97.8–98.1 % of the maximum absorbance could be reached after 4 h, as shown on Figure 6.

By using MOPS buffer, the maximum signal was reached after 6 h with an Al concentration of 85.1 ng/g, and after 8 h with an Al concentration of 265 ng/g, but 97.4–98.7 % of the maximum value is already reached after 2 h. In the case of Tris buffer, with an Al concentration of 81.3 ng/g, the measured signal fluctuated around the maximum with deviations below 0.01 absorbance change, between 6 hours and 3 days, while at an Al concentration of 208 ng/g, the maximum signal was reached after 7 h. In this case, we measured 99.2 % of the maximum value after 4 h. Accordingly, the waiting time is at least 2 h for MOPS buffer and at least 4 h for HEPES and Tris buffer. Afterwards, the measurement can be carried out at any time during the same working day (within 8 h). More details are provided in the Supporting Information.

**Figure 7:**
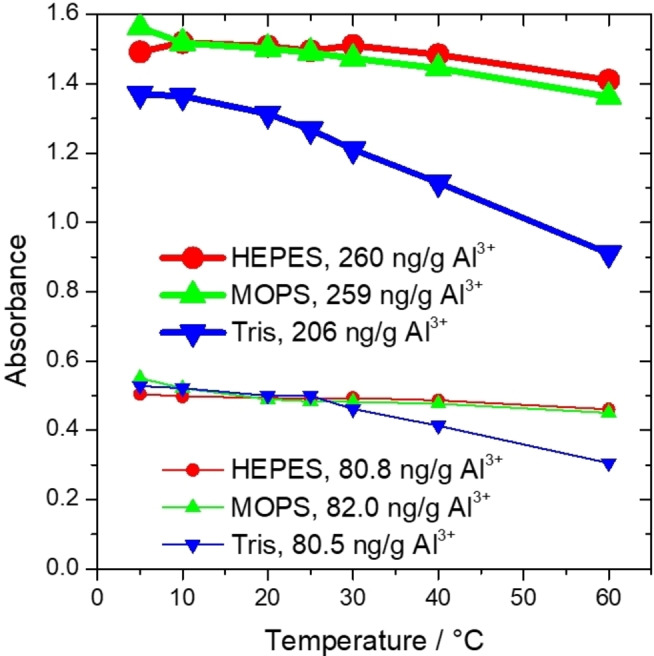
Temperature dependence of the analytical signal, examining the saturated NaCl medium.

To subsequent evaluation of the robustness of the proposed method, the temperature‐dependent sensitivity using different biological buffers was monitored (Figure 7).

It was revealed that while the temperature dependence of the absorbance is negligible in the case of HEPES and MOPS, the Tris‐containing system showed significant sensitivity to temperature change.

In the case of HEPES, slightly higher (1.5–1.6 %) absorbance values were observed by changing temperature from 25 to 10 °C. When temperature increased from 25 to 60 °C, the absorbance decreased significantly by 5.7–6.0 %. In the case of MOPS, a similar change was observed in 10–60 °C. In the case of Tris, the significantly higher temperature dependence was observed in 5–60 °C. Accordingly, the slight change in the absorbance measurement temperature may cause minor (<3 %) errors for HEPES and MOPS. Due to the higher temperature sensitivity, the precise calibration and measurement temperature is highly desired for Tris. Consequently, the use of HEPES‐ and MOPS‐based systems could be recommended when room temperature is used for Al‐determination. Noteworthy that the inverse effect of temperature on the measured absorbance was detected in the case of all proposed biological buffers compared to the effect observed for acetate‐containing system at pH=5.^[58]^ In terms of temperature adjustment, the measurement could be conveniently made by the relatively wide time frame (2–4 h) between the sample preparation and absorbance measurement. During this time, leaving the sample at room temperature, its temperature stabilizes to the room temperature at which the calibration was performed, without any other procedure. If this does not occur, a correction function can be used to correct the measured absorbance to the absorbance at the calibration temperature. The fact that the Al content did not significantly affect the percentage of change in absorbance value in response to a given temperature change should be noted as an advantage of this method.

It can be assumed that temperature could have direct and indirect effects on the detected signal as a synergistic result of several intrinsic properties. It is well‐known that the temperature has effect on the equilibrium constants *via* the temperature dependence of water ionization and reaction rates of complex formation.^[78]^ Furthermore, the change in temperature can hence change the species distribution of complex ions in the system, which, as described above for the pH effect, can lead to different solution absorbance due to the different spectra of each complex ion.

The analytical performance characteristics were also investigated for tested biological buffers. The detection and the quantification limits were determined on the basis of the 3‐fold and 10‐fold standard deviation of the blank signal. The sensitivity of the measurement is 2/3 of the slope of the calibration line. The linear range of the measurement was given in accordance with the quantification limit and the well‐known linearity limit of 2 of the Lambert‐Beer law. The obtained analytical performance characteristics and the calculated molar absorption coefficients (*ϵ*) for each buffer are presented in Table 1. The average density of the mixed solutions was determined as 1.12 g/cm^3^ and it was used for the calculation of *ϵ*.

**Table 1:** Analytical performance characteristics of the measurement method using biological buffers, examining the saturated NaCl medium.^[a]^

Entry		**HEPES**	**MOPS**	**Tris**
**1**	**Limit of detection**	1.8 ng/g	2.0 ng/g	1.9 ng/g
**2**	**Limit of quantification**	5.9 ng/g	6.8 ng/g	6.3 ng/g
**3**	**Sensitivity (*S*)**	3.846 (μg/g)^−1^	3.911 (μg/g)^−1^	4.001 (μg/g)^−1^
**4**	**Molar absorption coefficient (*ϵ*)**	$1.39\cdot 10^{5}$ Lmol^−1^cm^−1^	$1.42\cdot 10^{5}$ Lmol^−1^cm^−1^	$1.45\cdot 10^{5}$ Lmol^−1^cm^−1^
**5**	**Linear range**	5.9–469 ng/g	6.8–470 ng/g	1.9–349 ng/g

^[a]^ With an exception of the molar absorption coefficient, the data refer to the original sample before dilution with the assay solution and the buffer‐surfactant solution.

Compared to the acetate‐based system, 32.8–38.1 % increase in sensitivity could be obtained depending on the applied (entry 3). However, no significant differences were observed; quantification limits were found in the 5.9–6.8 ng/g interval (entry 2). It should be noted that both standard deviations for the blank's signal (0.0023–0.0027) and sensitivity values (3.85–4.00 (μg/g)^−1^) were almost the same for all the tested biological buffers. While the linear range was almost the same for HEPES and MOPS, Tris gave linear correlation up to 349 ng/g, 26 % less than others (entry 5).

**Figure 8:**
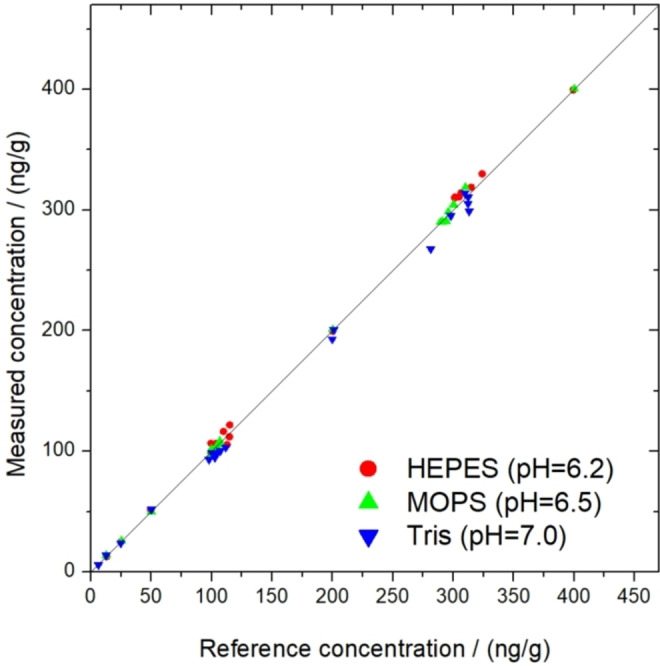
Validation of the measurement methods – the measured dissolved aluminum concentration as a function of the real concentration of standard solutions for all three tested biological buffers. Thin black line shows the ideal case, when the measured and reference concentrations are equal.

The improved measurement method was validated by measuring of standard samples with known concentrations. For all three buffer systems, 18 reference solutions were re‐measured for each buffer in their dynamic ranges. In the case of concentrations around 100 and 300 ng/g, several parallel reference solutions were remeasured confirming the method‘s precision. The differences between the measured and reference concentrations are summarized on Figure 8.

It was shown that the proposed measurement method was found to be suitable having high accuracy for all introduced buffer systems. The average of the differences between the test and reference sample was 2.9 %. The method was also validated with real samples from chlor‐alkali electrolysis by comparison with the ICP‐AES method. In the examined 10 cases, the difference between the results of the two methods was less than 10 %. Consequently, the proposed method enables a very accurate and facile measurement, even in the case of small dissolved aluminum concentrations. The detailed statistical data series are presented in Supporting Information.

## Conclusions

It can be concluded that our original Al determination method in concentrated brine solutions could be improved by the introduction of HEPES, MOPS, and Tris biological buffers. The sensitivity of the measurement was increased by 32.8–38.1 % applying pH 6.2–7.0. In addition, the sensitivity alters much less with changes in salinity than in the case of an acetate buffer system, which allows an informative measurement to be performed without salinity determination and calibration. The only disadvantage of the biological buffers applied is the longer waiting time required to reach the appropriate analytical signal. In all other aspects, the buffers tested (HEPES, MOPS, Tris) provide a more efficient determination of Al.

While a moderate sensitivity of the measurements for temperature change was observed, our method remained relatively robust in the temperature range of 10–60 °C applying HEPES or MOPS buffers, as opposed to the case of Tris. Furthermore, a significantly wider dynamic range can be achieved using HEPES or MOPS buffers than applying Tris buffer. The use of MOPS is also supported by the fact that the obtained optimal pH and the p*K*_a_ value of the buffer are the closest to each other of the tested biological buffers. Overall, based on these findings, the use of the MOPS buffer is the most recommended.

The developed method could be used in an industrial environment and provide a fast, robust, and facile method for determining Al‐content in a concentrated brine solution at ppb level. Thus, there is no need for expensive mass spectrometry‐based analytical methods.

## Experimental Section

### Apparatus and Chemicals

Absorbance and pH measurements were performed with a Specord 210 Plus UV‐VIS (Analytik Jena, Jena, Germany) spectrophotometer and a Mettler Toledo (Columbus, Ohio, USA) FiveEasy instrument, respectively. The temperature of the solutions was also continuously monitored via in‐built sensors of the FiveEasy instrument. The reference ICP‐AES measurements were made with an Spectro Arcos II. (Kleve, Germany) instrument. The weights required for analytical accuracy were measured with a Sartorius (Göttingen, Germany) Quintix224‐1 CEU digital scale.

Sodium chloride (99.9 %), solid aluminum (99.8 %), ECR (pure), CTAB (>99 %), buffer solutions including anhydrous acetic acid 99.9 %), sodium acetate trihydrate (100.0 %), Tris, concentrated HCl solution (analytical grade), MOPS (ultra‐pure–>98.5 %), sodium hydroxide and the sodium salt of HEPES were obtained from VWR International Kft. (Debrecen, Hungary) and used as received.

The solutions were prepared according to standard laboratory methods.

### Preparation of Aluminum Standard and Buffer Solutions

The standard solution of Al was prepared by measuring solid analytical grade aluminum and dissolving it in 5 M hydrochloric acid. Our standard solutions for calibrations were prepared by dilution of Al stock solution with distilled water, or 5, 10, 15, 20, 27 wt% NaCl solution to adjust the desired concentration in the ppb range. Because glass tools affect the aluminum content of the solutions via adsorption and desorption phenomena,^[21]^ plastic equipment was used in our experiments.

Buffer‐surfactant and ECR solutions were prepared by weighing the reactants using an analytical scale and then dissolving reactants with distilled water in a volumetric flask. ECR solutions contained 0.08000 wt% of the reactant, adjusting the pH to 2.9 using acetic acid. During the preparation of buffer‐surfactant solutions, the obtained pH value was checked by independent pH measurements. Three different buffer solutions were made, and a smaller number of solutions were added to the samples; the buffers contained 0.546 w% CTAB, too. These are:

– Tris‐HCl (22.5 wt% Tris; pH=7.0 with HCl solution),
– MOPS‐NaOH (13.5 wt% MOPS; pH=6.5 with NaOH solution) and
– HEPES‐NaOH (18 wt% HEPES sodium salt; pH=6.2 with NaOH solution).

The pH was adjusted by the addition of the appropriate amounts of 20 and 5 wt% HCl or NaOH solution during simultaneous measurements.

When measuring the solutions, 15.0 g sample, 2.5 g ECR, and 5.0 g CTAB‐buffer mixed solution were added. The waiting time between adding reactants and the absorbance measurement depended on the buffer. The results show that for the acetate buffer, the corresponding measurement time window was a 15–90 min interval.^[58]^ For MOPS the minimal waiting time was ca. 2 h and 4 h for Tris and HEPES, respectively. The measurements were completed at the beginning of the time window with constant absorbance to obtain comparable acquired data sets.

## Supporting Information

The Supporting Information provides more details about the effect of salinity on the optimal wavelength of the measurement and the effect of time on the absorbance in a time interval of 3 days. Moreover, it contains the measurement data of validation of the method and spectra of the standard solutions used as the basis of Figure 3, Figure 4 and Figure 5.

## Author Contributions

BC: conceptualization, experimental work, data acquisition, handling and analysis, writing and editing of the manuscript, funding acquisition. LF: conceptualization, methodology. MC: experimental work, data acquisition. LTM: Writing of the final version of the manuscript, language editing, data analysis, and interpretation. ILG: funding acquisition, supervision.

## Conflict of Interests

No potential conflict of interest was reported by the authors. The authors declare that they have no known competing financial interests or personal relationships that could have appeared to influence the work reported in this paper.

1

## Supporting information

As a service to our authors and readers, this journal provides supporting information supplied by the authors. Such materials are peer reviewed and may be re‐organized for online delivery, but are not copy‐edited or typeset. Technical support issues arising from supporting information (other than missing files) should be addressed to the authors.

Supporting Information

## Data Availability

The data that support the findings of this study are available from the corresponding author upon reasonable request.
