# Supplementary material for: Facile Determination of Aluminum Content in Industrial Brine by Investigating the Effects of Buffer Systems
Source: ChemistryOpen. 2024 Sep 3;13(12):e202400038. doi: 10.1002/open.202400038 (PMC11625942; doi:10.1002/open.202400038)
Supplement: Supplementary file 1 — Supporting Information [file OPEN-13-e202400038-s001.pdf]

ChemistryOpen

Supporting Information

Facile Determination of Aluminum Content in Industrial Brine by Investigating the Effects of Buffer Systems

Benjámin Csorba, László Farkas, Marcell Csécsi, László T. Mika, and Iván L. Gresits*

Facile Determination of Aluminum Content in Industrial Brine by Investigating the Effects of Buffer Systems

Benjámin Csorba,[a, b] László Farkas,[b] Marcell Csécsi,[b] László T. Mika,[a] and Iván L. Gresits*[a]

[a] B. Csorba, Dr. L. T. Mika, Dr. I. L. Gresits
Department of Chemical and Environmental Process Engineering, Faculty of Chemical Technology and Biotechnology
Budapest University of Technology and Economics
Műegyetem rkp. 3., H-1111 Budapest, Hungary
E-mail: gresits.ivan@vbk.bme.hu

[b] B. Csorba, L. Farkas, M. Csécsi
Process Technology Support
BorsodChem Ltd.
Bolyai tér 1., H-3700 Kazincbarcika, Hungary

Supporting Information

Table of content

1. **Effect of the salinity on the optimal wavelength using each buffer system**
2. **Effect of time on the analytical signal for each buffer system – more details**
3. **Validation dataset for each buffer system**
4. **Spectra of standard solutions using the examined buffer systems**

1. **Effect of the salinity on the optimal wavelength using each buffer system**

The salinity had an influence on the optimal wavelength of the measurement. These data are shown in Table S1.

Table S1. The optimal measurement wavelength as a function of salinity according to two approaches (all wavelengths are in nm unit). The source of the acetate buffer data in case of concentrated saline medium is our own previous article.[1]

NaCl-content / wt%	Fit with the highest sensitivity				Fit with the highest R^2			
	Ace-tate	HEPES	MOPS	Tris	Ace-tate	HEPES	MOPS	Tris
0	589	608	608	617	603	596	596	620
5	596	606	606	615	600	597	595	617
10	595	605	604	614	600	594	597	611
15	594	600	600	609	602	591	596	607
20	594	599	599	610	607	594	596	607
27 (conce-ntrated)	596	599	599	610	597	594	596	610

Based on Table S1, at low salinity, in the case of HEPES and MOPS, the maximum sensitivity could be detected at a significantly higher wavelength than the best fit, however, as the salinity increased, this difference decreased, in concentrated saline media the difference is only 1-5 nm. This effect could be favorable, since in the optimal case, the two maxima belong to the same wavelength. In this case, the method would be as accurate and sensitive as possible, in the opposite case, a compromise must be made between the two aspects. Thus, increasing the salinity is expedient from this point of view.

2. **Effect of time on the analytical signal for each buffer system – more details**

It should be noted that however the absorbance was stable for at least 8 h (a working day) for all proposed buffer systems, the absorbance began to decrease later. This effect is shown in Figure S1.

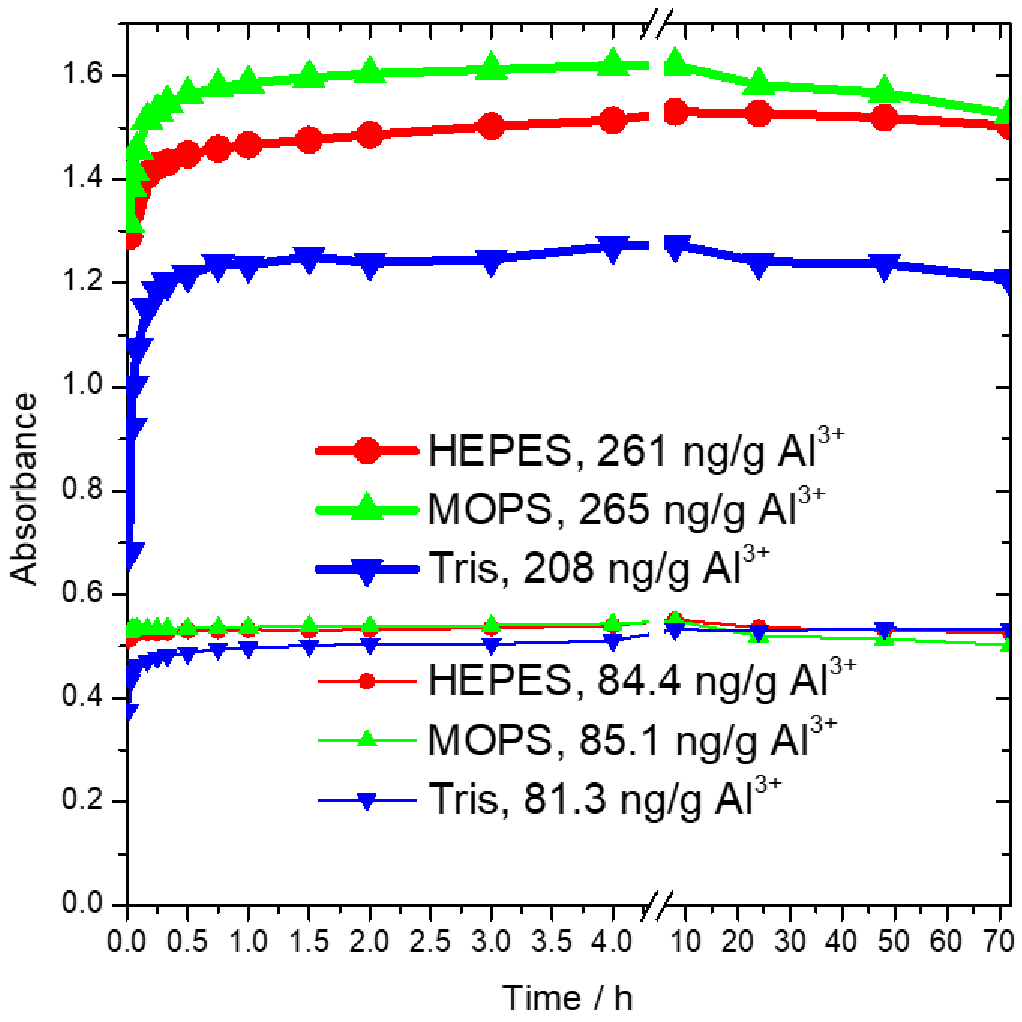

Figure S1. Time dependence of the analytical signal using different buffers for 3 days, examining a saturated NaCl medium.

By using MOPS buffer, 1 day after mixing the reactants, the measurable signal dropped to 95.3-96.1% of the maximum signal. In contrast, using HEPES-based buffer, after 30 h, 98.4–98.8% of the maximum absorbance was still measurable. In the case of Tris buffer, with Al concentration of 81.3 ng/g, the measured signal fluctuated around the maximum with deviations below 0.01 absorbance change, between 6 hours and 3 days, while at an Al concentration of 208 ng/g after 1 day the signal decreased to 96.8% of the maximum value. Based on what is described here, we recommend that if HEPES buffer is used, the absorbance measurement of the solutions be performed no later than the next day after adding the reactants, however, in the case of the other two buffers, on the same day.

3. Validation dataset for each buffer system

The validation data using the examined biological buffers is shown in Table S2. Fig. 8 of the original article is based on these data.

Table S2. Reference and measured concentrations for the validation of our proposed method using each buffer systems.

HEPES			MOPS			TRIS		
Reference concentration / (ng/g)	Measured concentration / (ng/g)	Relative deviation	Reference concentration / (ng/g)	Measured concentration / (ng/g)	Relative deviation	Reference concentration / (ng/g)	Measured concentration / (ng/g)	Relative deviation
99.5	106.5	7.0%	103.7	100.8	2.8%	107.2	100.4	6.3%
306.4	314.2	2.5%	295.9	298.5	0.9%	309.7	314.1	1.4%
103.2	106.3	3.0%	102.3	99.3	2.9%	111.6	103.1	7.6%
301.4	310.7	3.1%	300.3	304.5	1.4%	298.0	295.3	0.9%
115.0	121.9	6.0%	106.8	108.3	1.4%	6.3	5.9	6.7%
305.0	311.0	2.0%	310.2	318.9	2.8%	281.5	267.8	4.8%
109.8	116.4	6.0%	400.3	400.5	0.0%	97.8	93.3	4.6%
301.3	309.9	2.9%	200.2	200.8	0.3%	102.7	94.7	7.7%
114.7	112.0	2.3%	99.6	97.6	2.0%	104.7	98.1	6.3%
112.8	105.5	6.5%	50.1	50.0	0.2%	312.4	310.7	0.5%
324.0	329.9	1.8%	25.4	25.8	1.5%	312.3	305.4	2.2%
314.9	318.9	1.3%	12.7	13.7	8.0%	313.2	299.0	4.5%
399.3	399.6	0.1%	103.8	103.7	0.1%	201.2	200.8	0.2%
200.2	199.7	0.3%	99.8	101.7	2.0%	100.3	98.8	1.5%
99.6	98.7	0.9%	105.7	105.5	0.2%	50.1	51.8	3.3%
49.6	51.2	3.2%	294.2	290.9	1.1%	24.9	23.8	4.3%
25.0	25.0	0.1%	289.4	290.0	0.2%	12.7	13.8	8.3%
13.1	12.7	3.0%	291.1	290.4	0.2%	199.9	193.0	3.4%

4. Spectra of standard solutions using the examined buffer systems

The following figures show the spectra of the calibrations for each buffer in the medium with different salt contents. These measurements were the basis of the investigation of the effect of salt content and wavelength on the sensitivity and the regression factor (Fig. 3, Fig. 4 and Fig. 5 in the original article).

The aluminum concentration in the standard solutions refers to the state after dilution with the ECR assay solution and the buffer-surfactant solution as we discussed in the Experimental section of our original article. The salt content refers to the original samples before dilution. All figures show blank-corrected absorbances of the standard solutions.

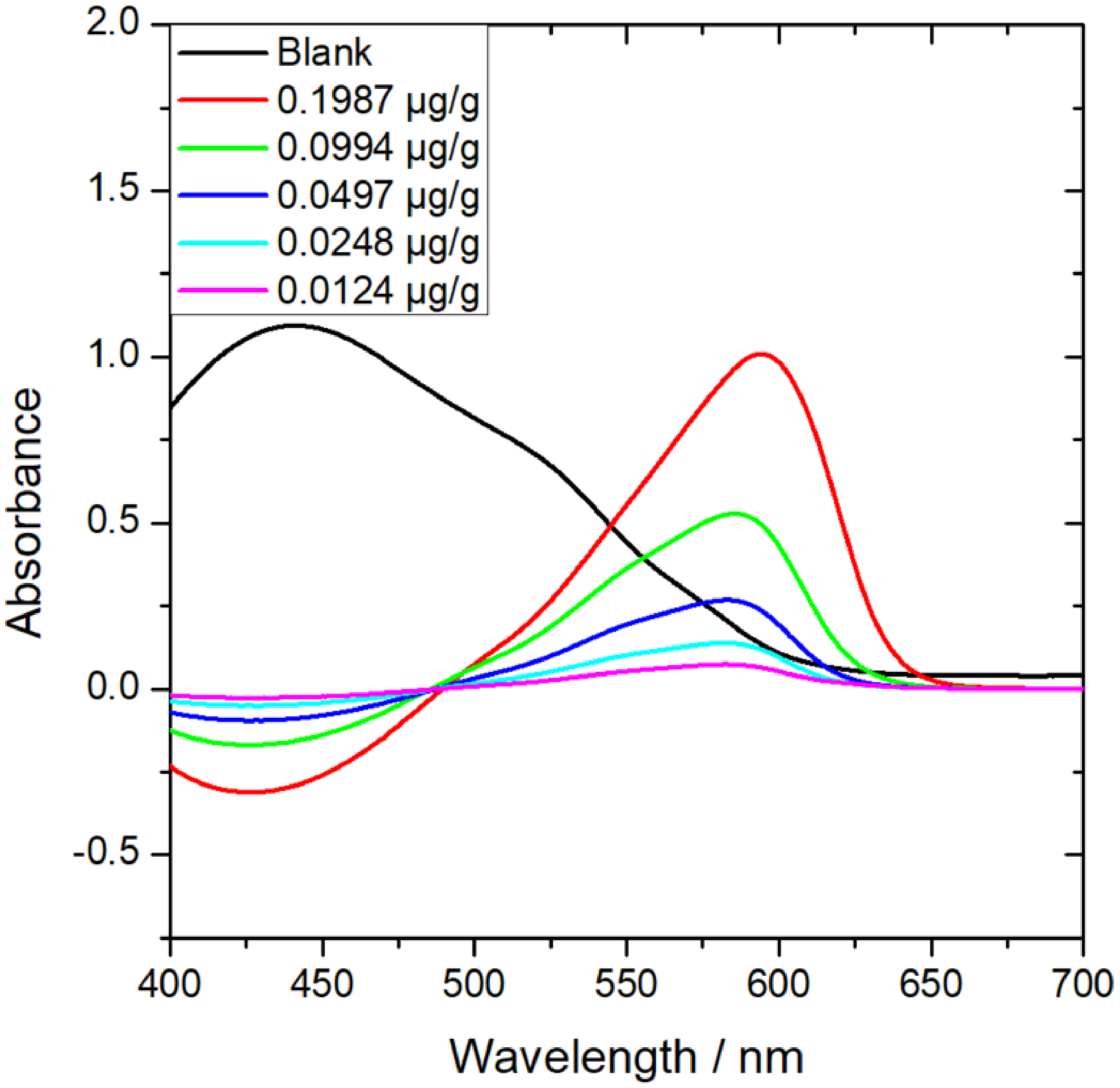

Figure S2. Spectra of the calibration solutions using HEPES buffer in a dilute aqueous medium.

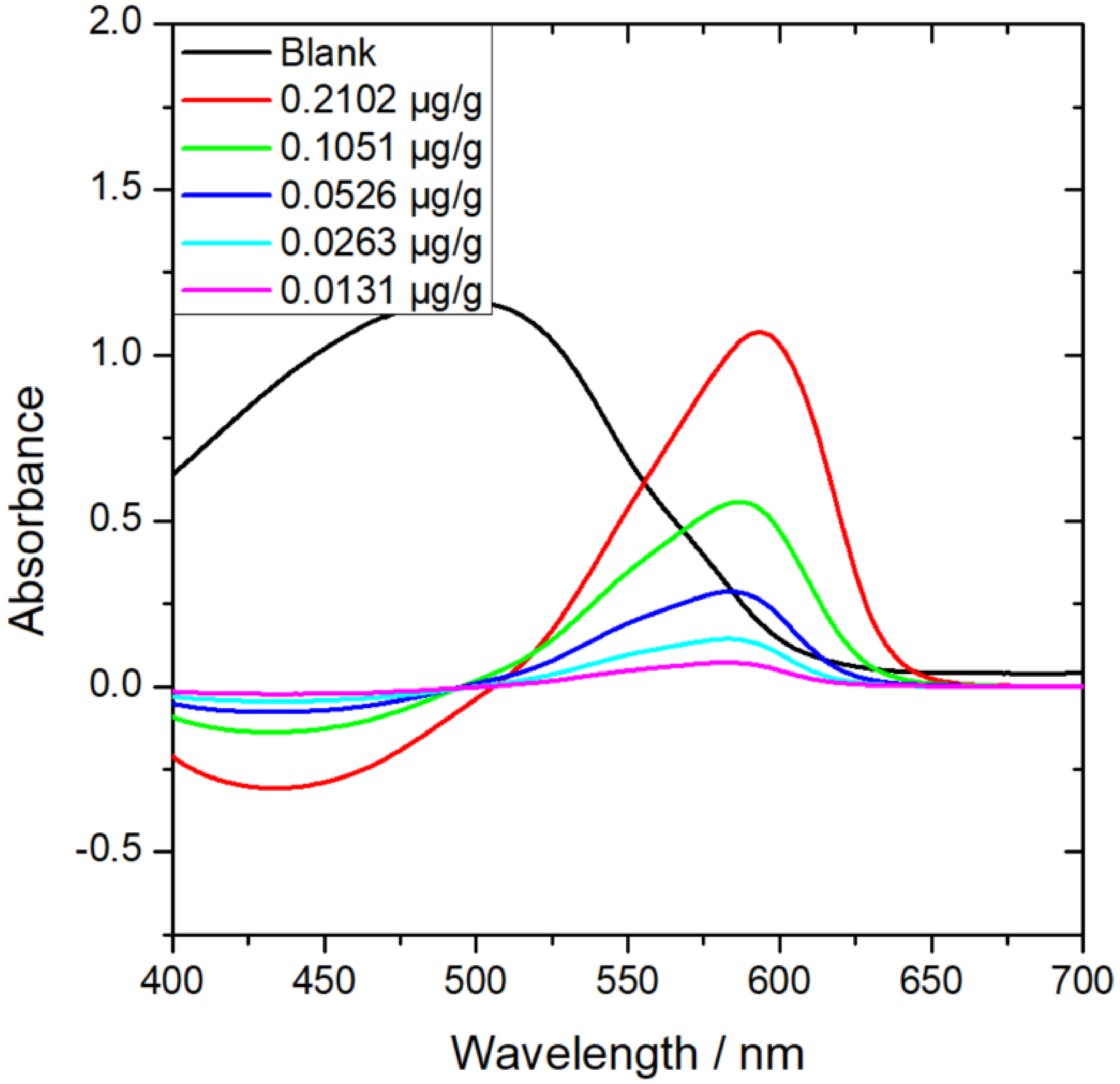

Figure S3. Spectra of the calibration solutions using HEPES buffer in a 5 wt% brine.

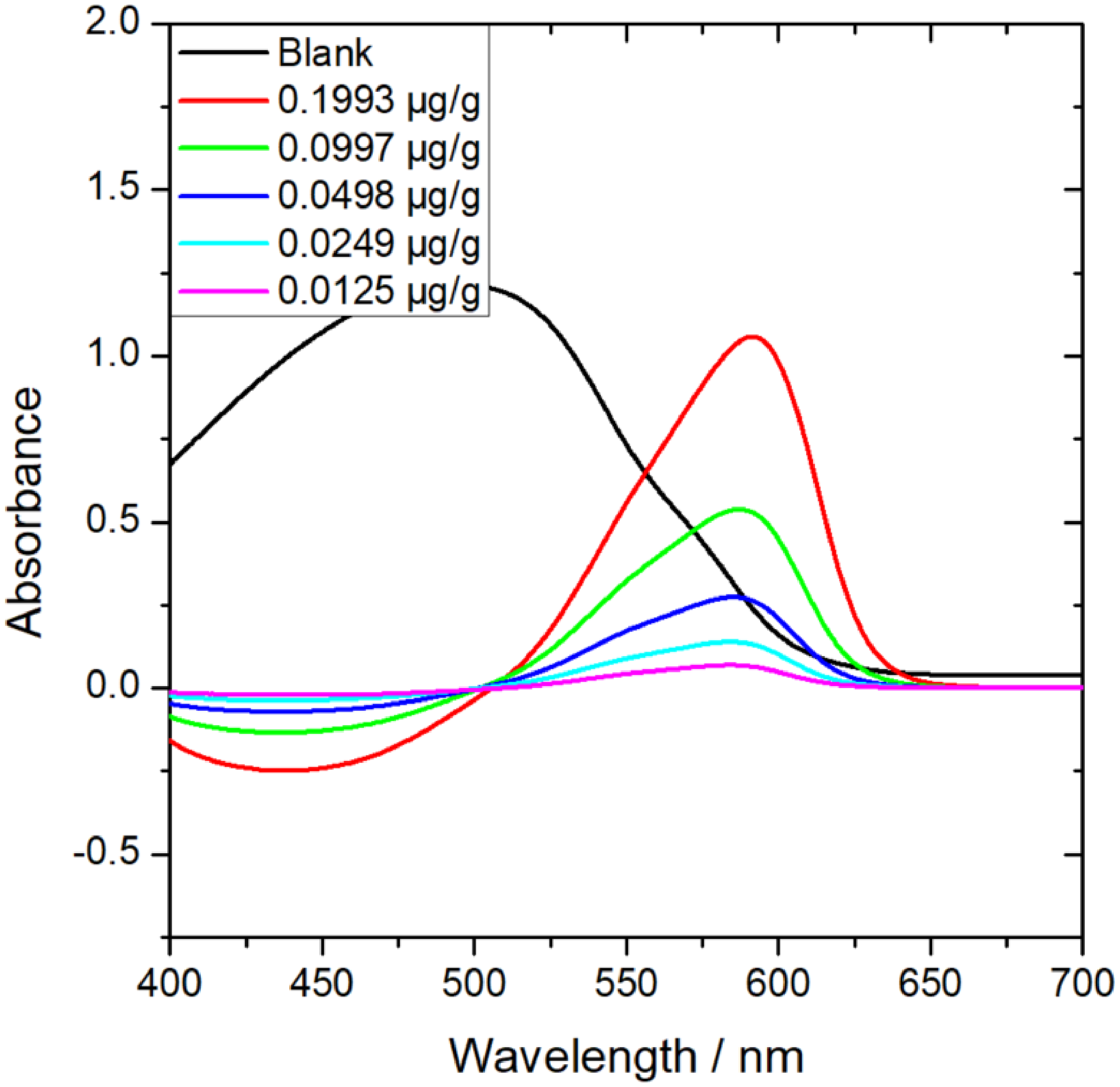

Figure S4. Spectra of the calibration solutions using HEPES buffer in a 15 wt% brine.

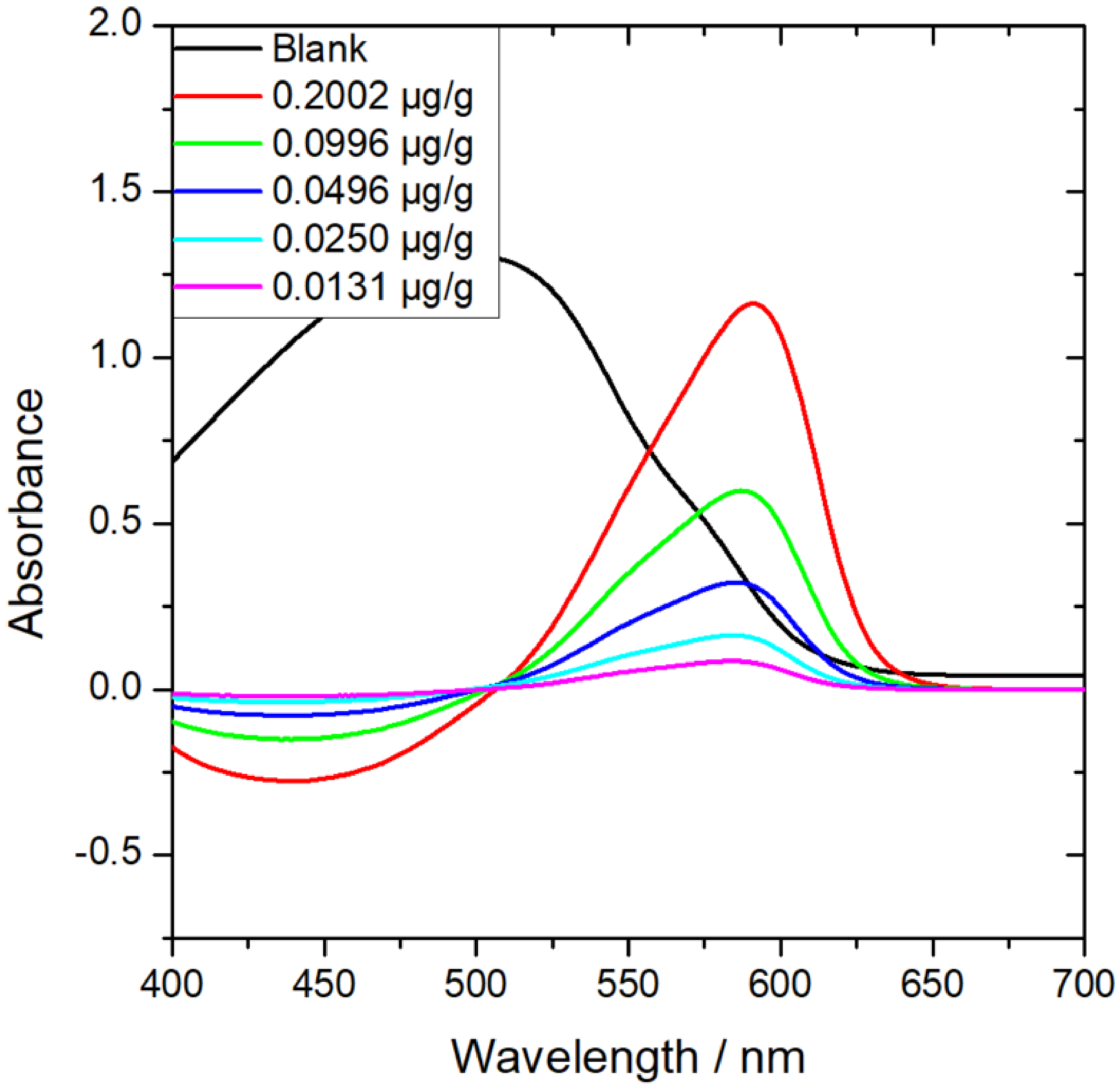

Figure S5. Spectra of the calibration solutions using HEPES buffer in concentrated brine.

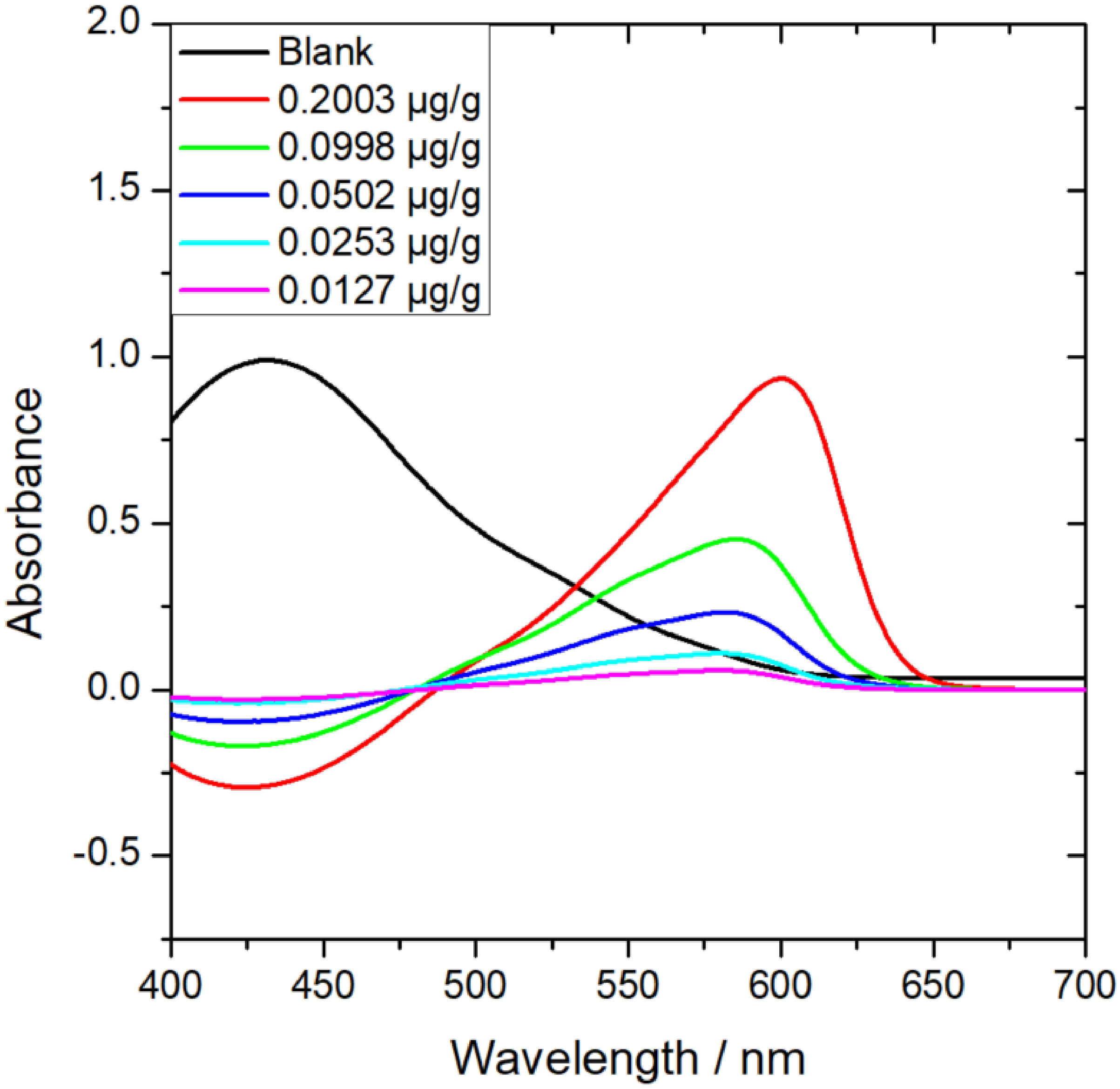

Figure S6. Spectra of the calibration solutions using MOPS buffer in a dilute aqueous medium.

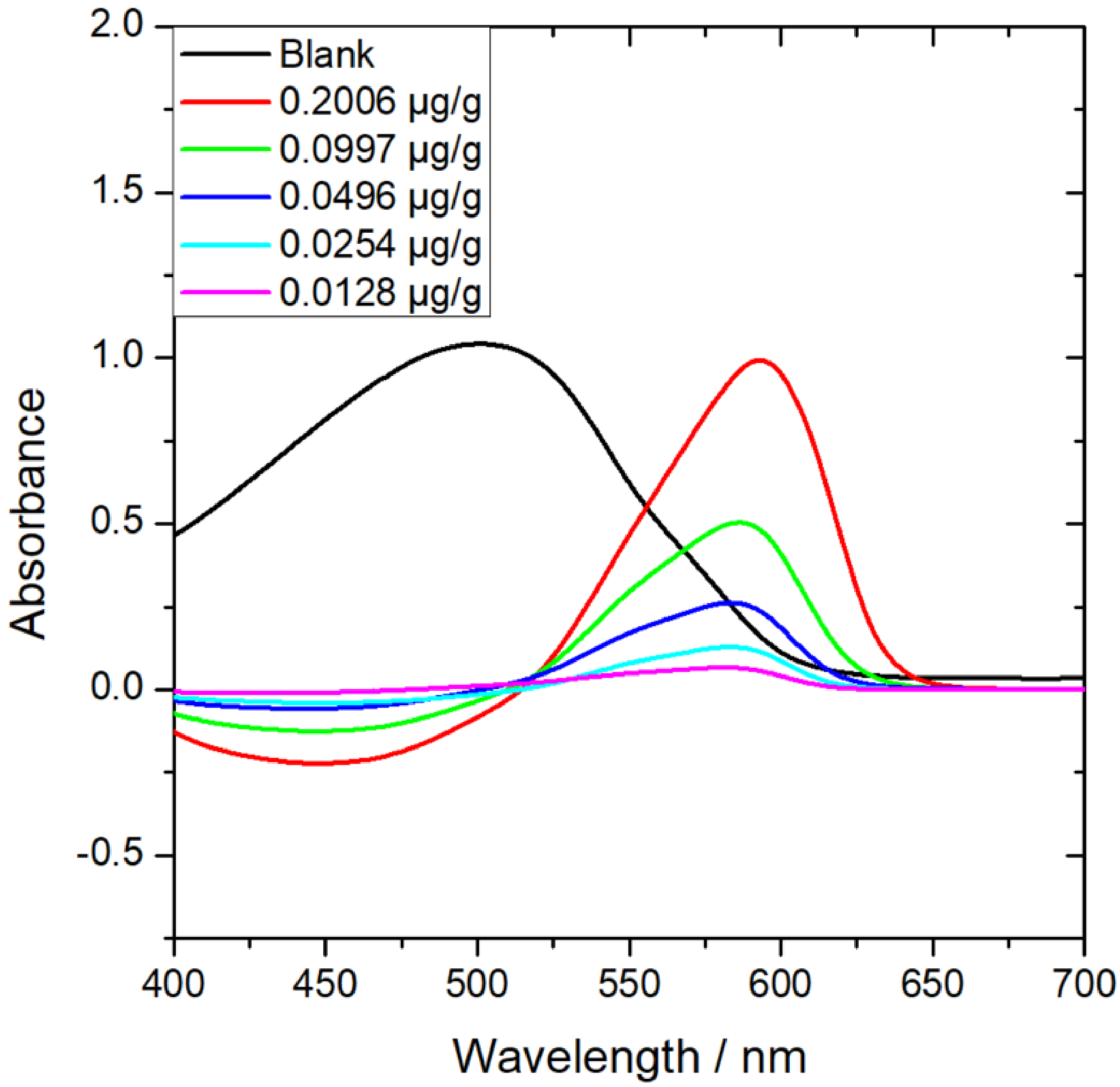

Figure S7. Spectra of the calibration solutions using MOPS buffer in a 5 wt% brine.

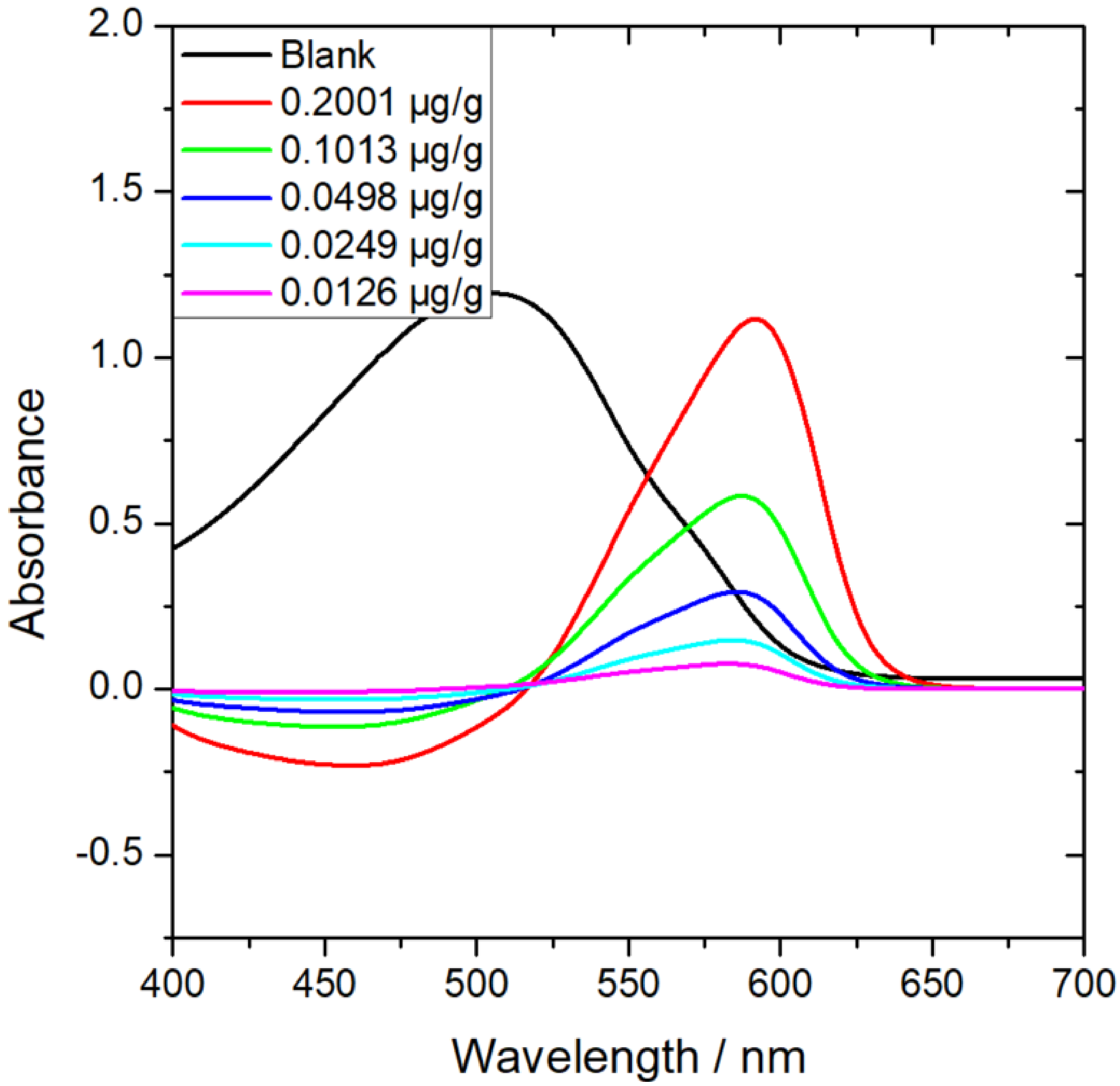

Figure S8. Spectra of the calibration solutions using MOPS buffer in a 15 wt% brine.

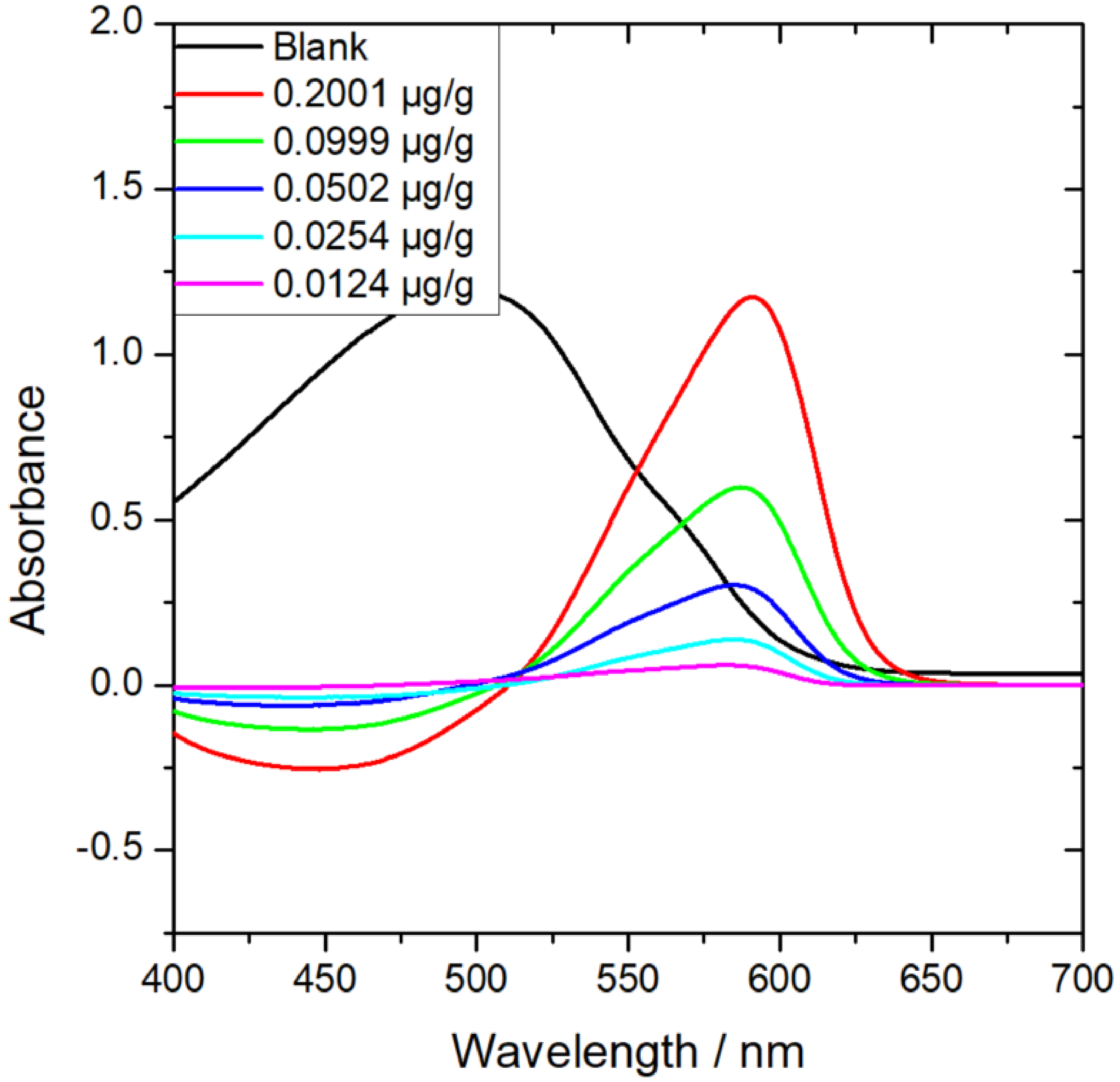

Figure S9. Spectra of the calibration solutions using MOPS buffer in concentrated brine.

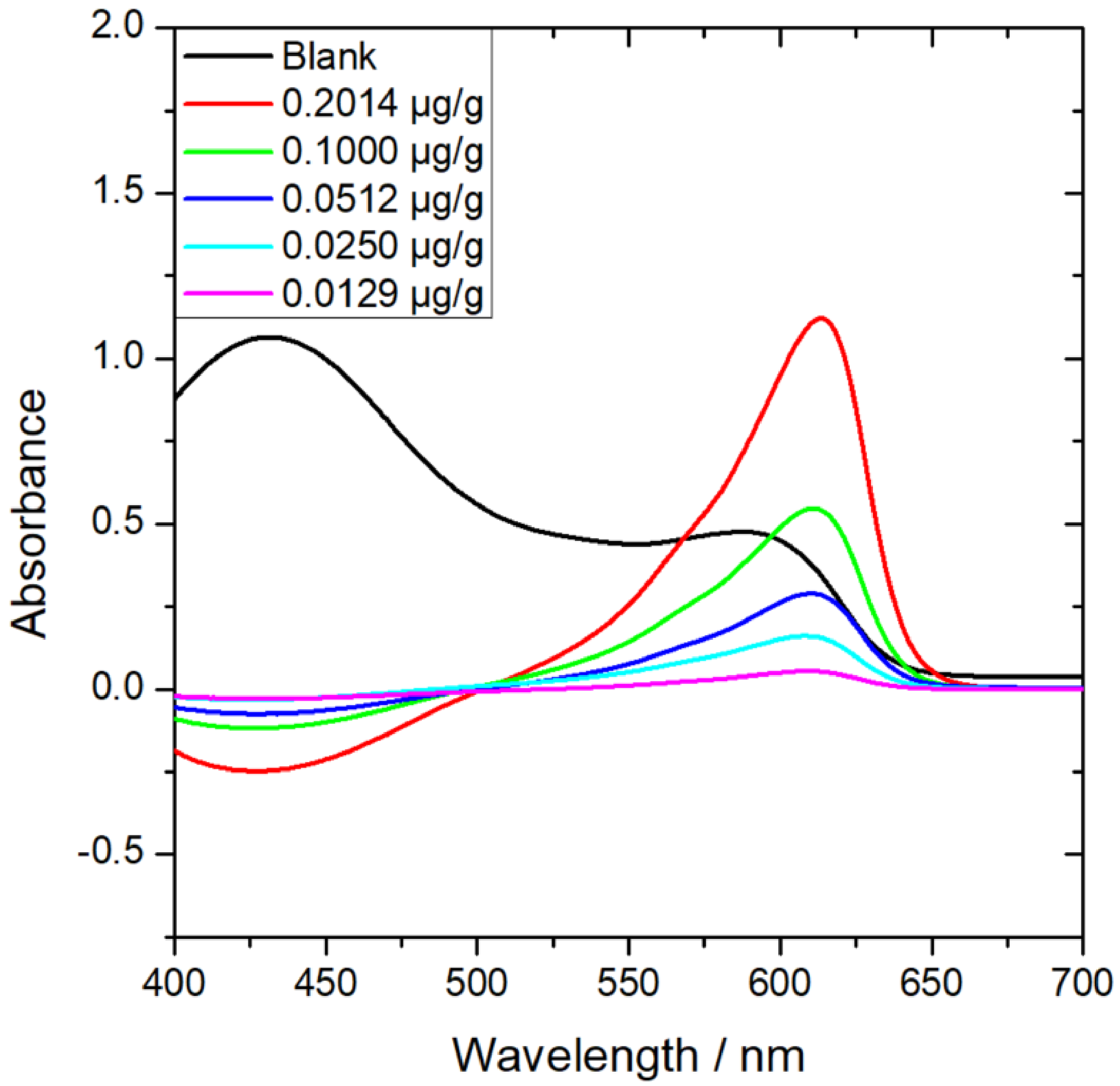

Figure S10. Spectra of the calibration solutions using Tris buffer in a dilute aqueous medium.

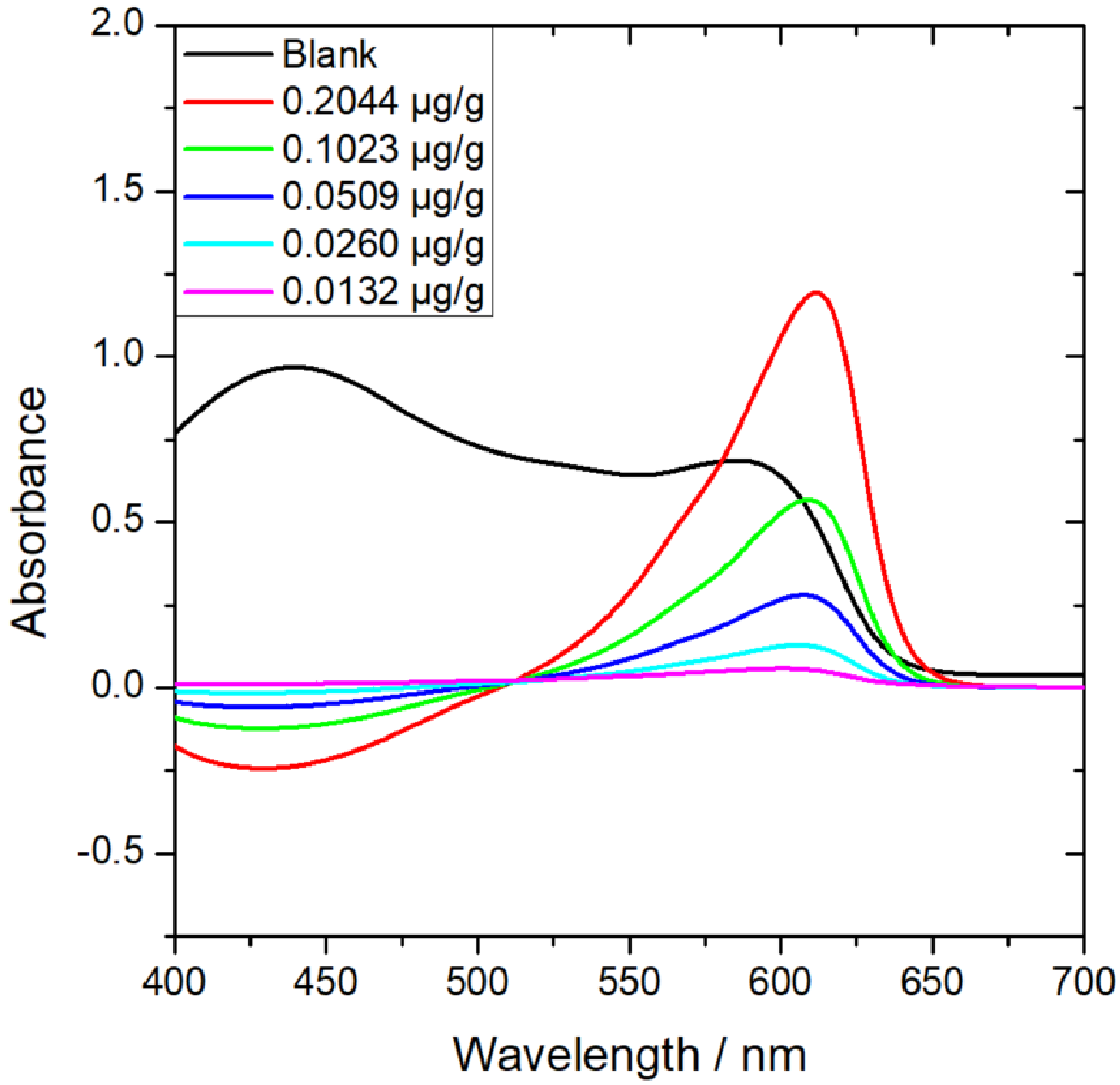

Figure S11. Spectra of the calibration solutions using Tris buffer in a 5 wt% brine.

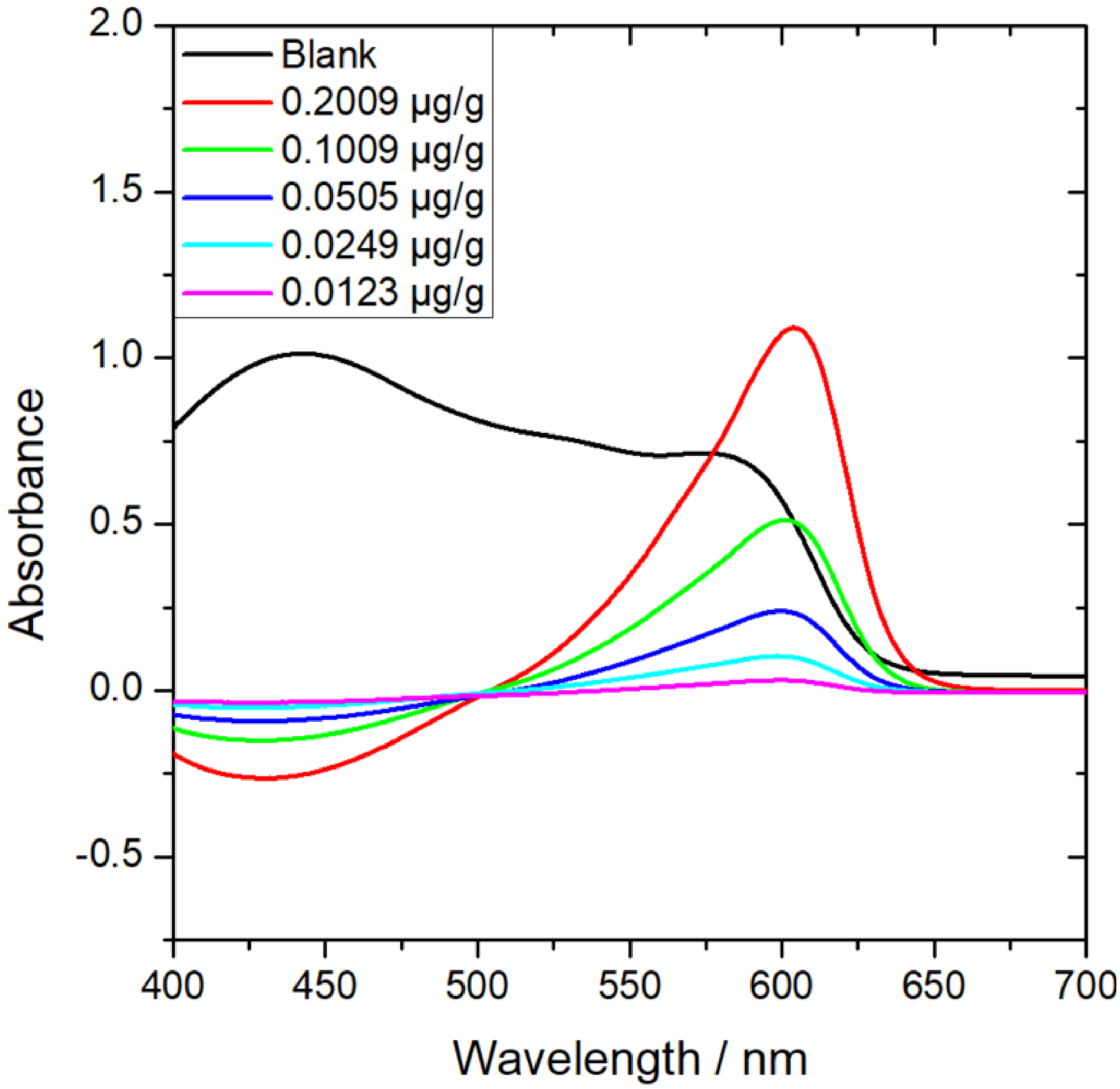

Figure S12. Spectra of the calibration solutions using Tris buffer in a 15 wt% brine.

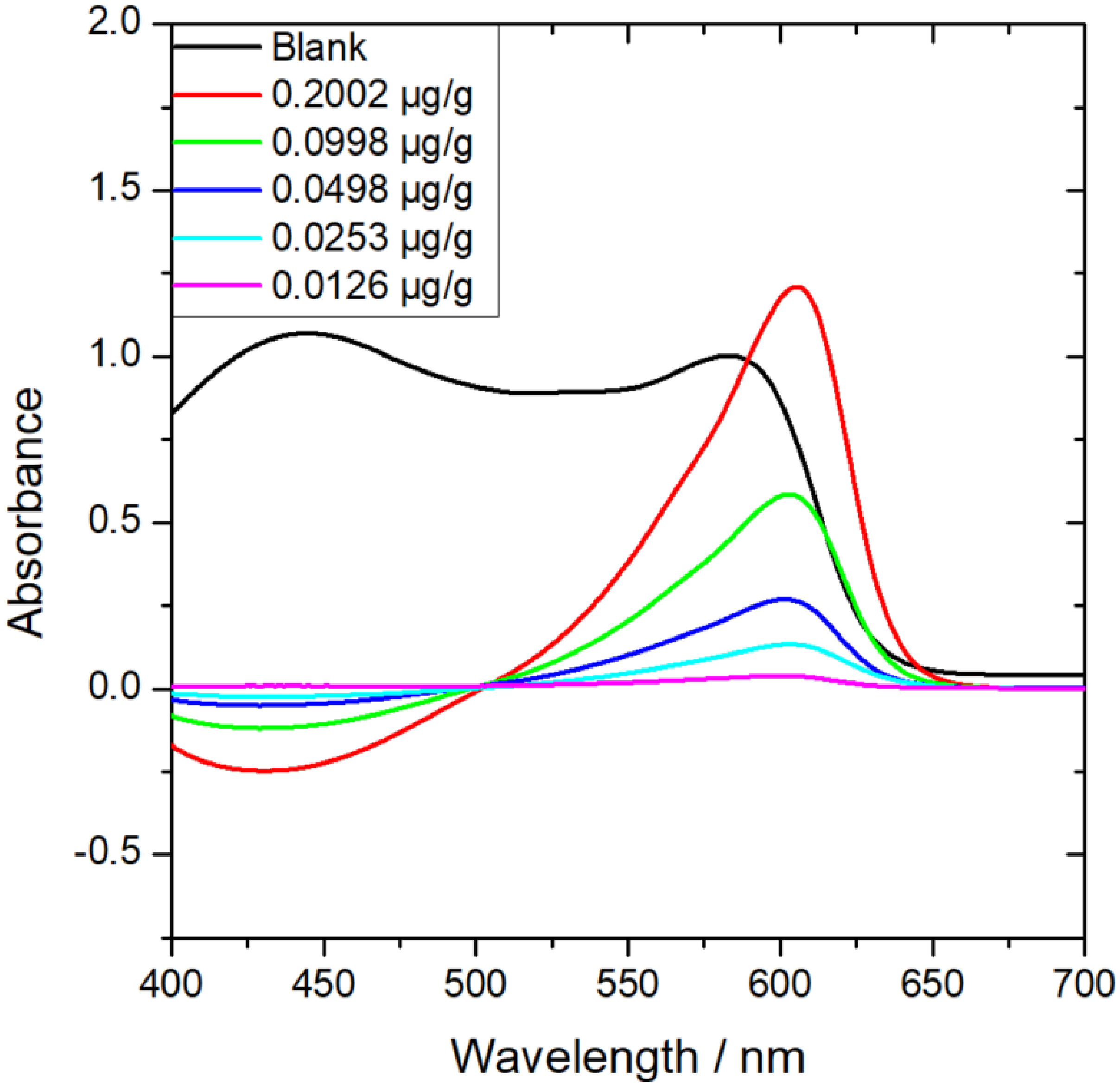

Figure S13. Spectra of the calibration solutions using Tris buffer in concentrated brine.

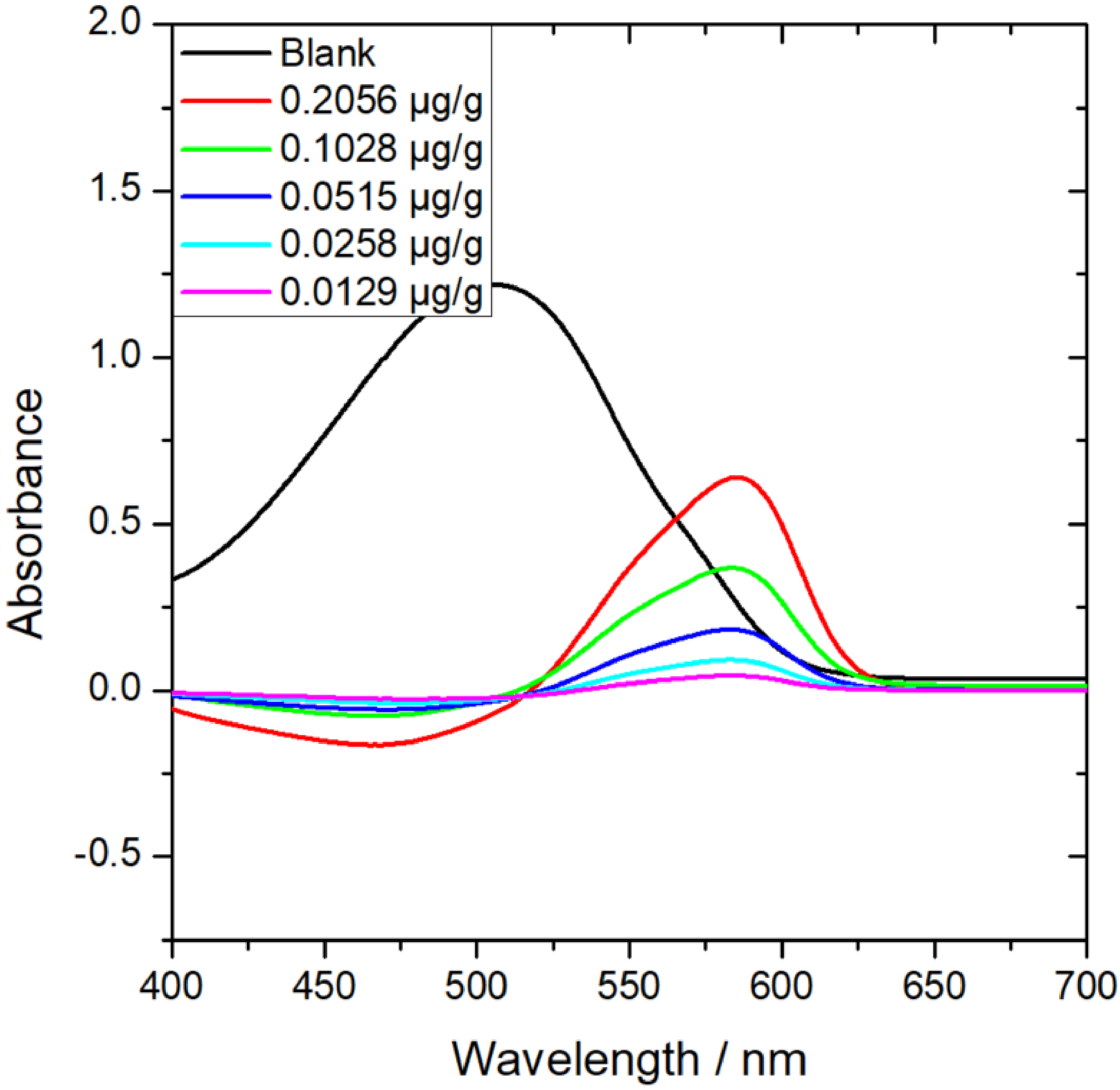

Figure S14. Spectra of the calibration solutions using acetate buffer in a dilute aqueous medium.

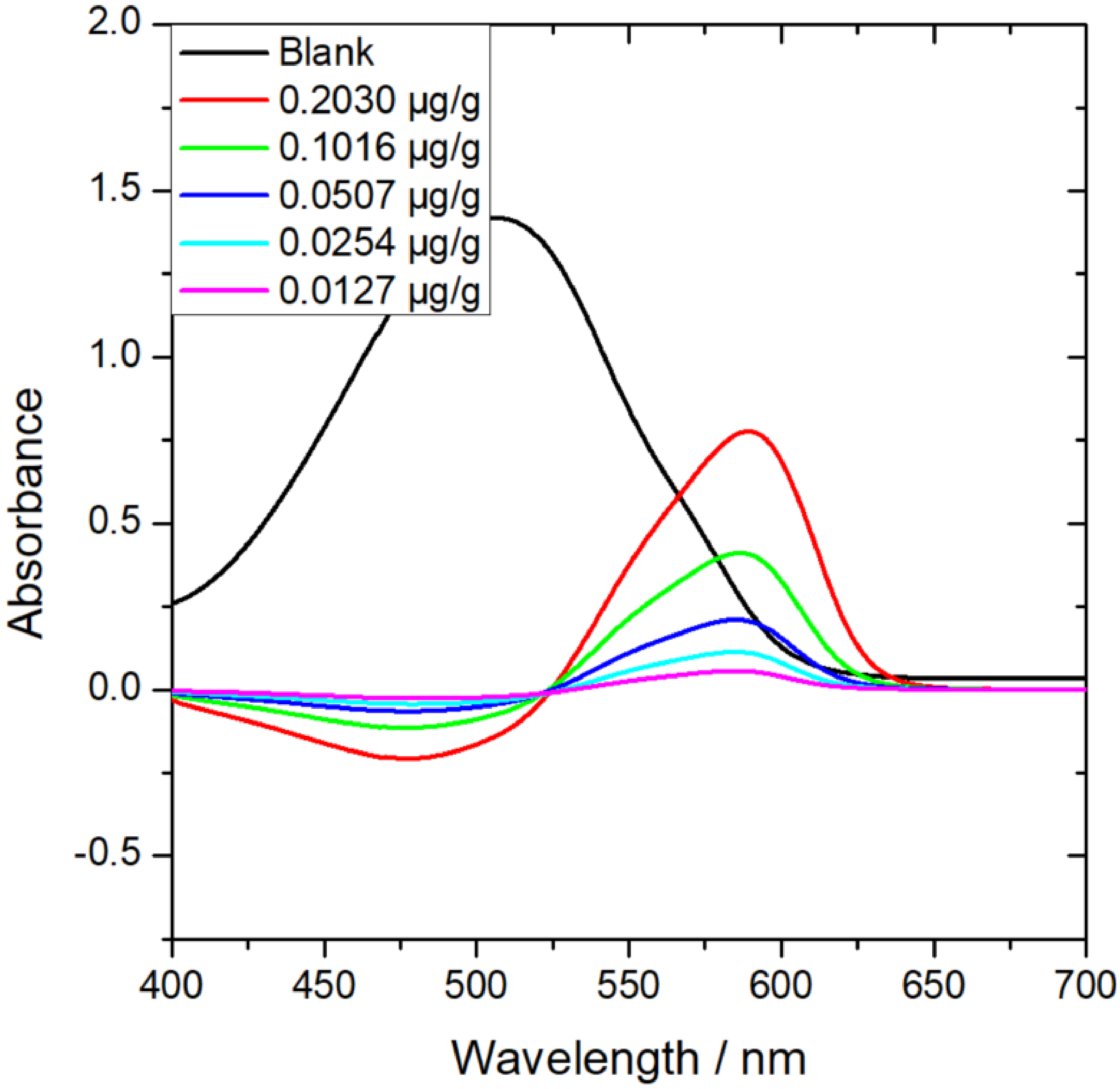

Figure S15. Spectra of the calibration solutions using acetate buffer in a 5 wt% brine.

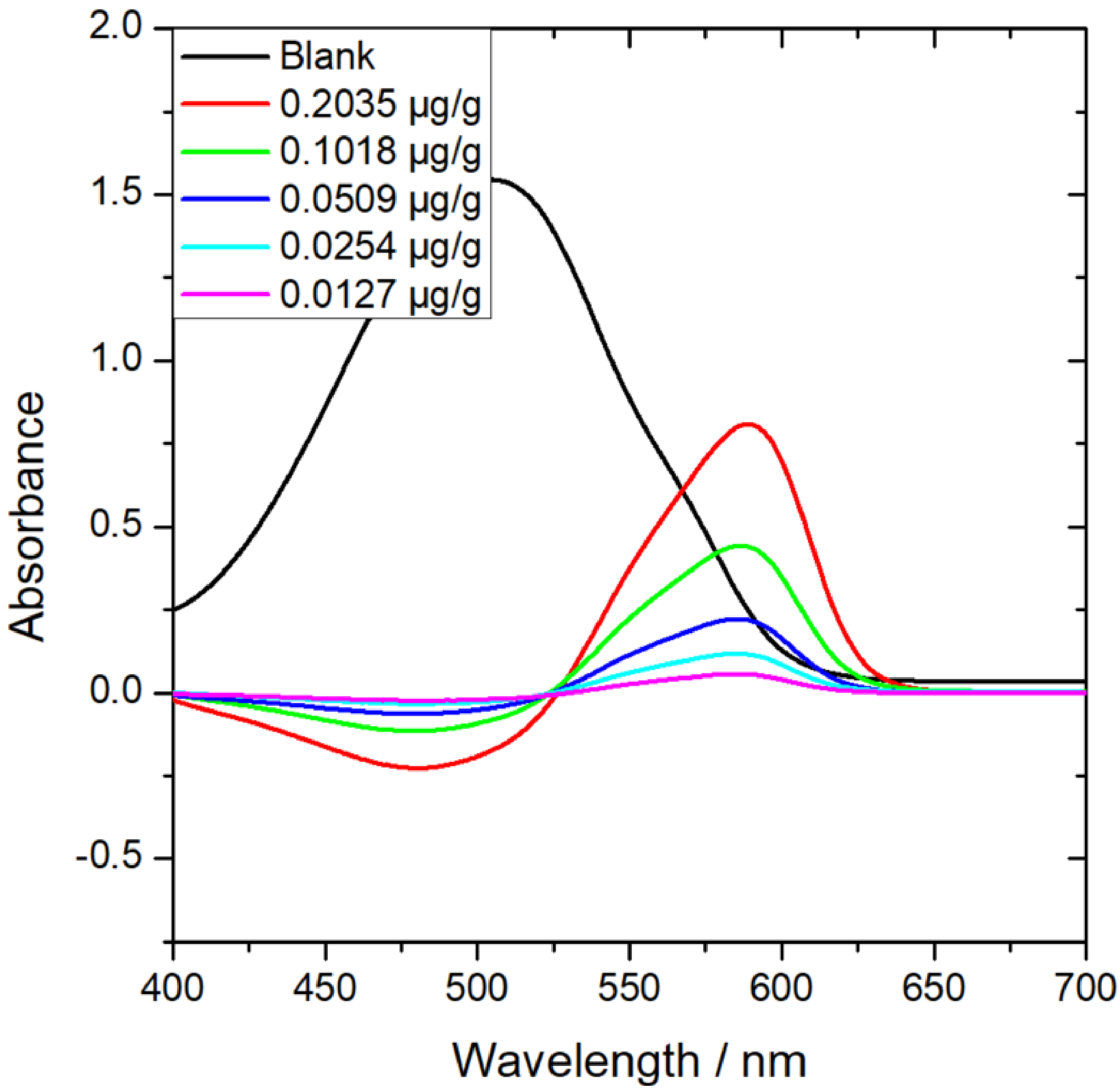

Figure S16. Spectra of the calibration solutions using acetate buffer in a 15 wt% brine.

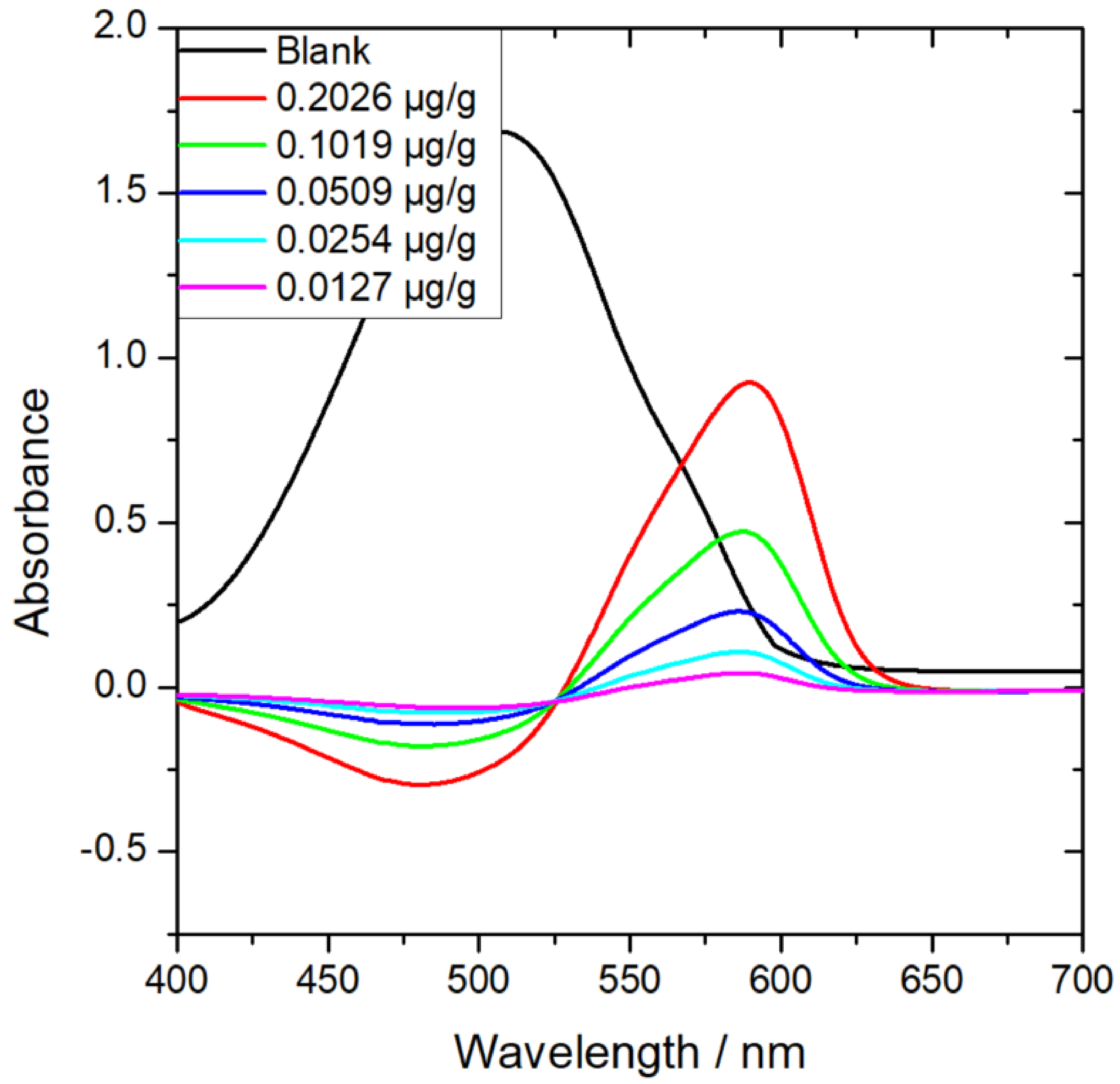

Figure S17. Spectra of the calibration solutions using acetate buffer in concentrated brine.[1]

References

[1] B. Csorba, L. Farkas, A. Mihalkó, R. Z. Boros, I. L. Gresits, *Period. Polytech., Chem. Eng.* **2023**, 67(3), 442-451.
